# Transcriptome analysis of two inflorescence branching mutants reveals cytokinin is an important regulator in controlling inflorescence architecture in the woody plant *Jatropha curcas*

**DOI:** 10.1186/s12870-019-2069-3

**Published:** 2019-11-04

**Authors:** Mao-Sheng Chen, Mei-Li Zhao, Gui-Juan Wang, Hui-Ying He, Xue Bai, Bang-Zhen Pan, Qian-Tang Fu, Yan-Bin Tao, Ming-Yong Tang, Jorge Martínez-Herrera, Zeng-Fu Xu

**Affiliations:** 1 CAS Key Laboratory of Tropical Plant Resources and Sustainable Use, Xishuangbanna Tropical Botanical Garden, The Innovative Academy of Seed Design, Chinese Academy of Sciences, Menglun, Mengla, 666303 Yunnan China; 2 Center of Economic Botany, Core Botanical Gardens, Chinese Academy of Sciences, Menglun, Mengla, 666303 Yunnan China; 3 College of Life Sciences, University of Chinese Academy of Sciences, Beijing, 100049 China; 4 Instituto Nacional de Investigaciones Forestales, Agrícolas y Pecuarias, Campo Experimental Huimanguillo, Huimanguillo, Tabasco Mexico

**Keywords:** Branching, Cytokinin, Inflorescence, Physic nut, WGCNA analysis

## Abstract

**Background:**

In higher plants, inflorescence architecture is an important agronomic trait directly determining seed yield. However, little information is available on the regulatory mechanism of inflorescence development in perennial woody plants. Based on two inflorescence branching mutants, we investigated the transcriptome differences in inflorescence buds between two mutants and wild-type (WT) plants by RNA-Seq to identify the genes and regulatory networks controlling inflorescence architecture in *Jatropha curcas* L., a perennial woody plant belonging to Euphorbiaceae.

**Results:**

Two inflorescence branching mutants were identified in germplasm collection of *Jatropha.* The *duo xiao hua* (*dxh*) mutant has a seven-order branch inflorescence, and the *gynoecy* (*g*) mutant has a three-order branch inflorescence, while WT *Jatropha* has predominantly four-order branch inflorescence, occasionally the three- or five-order branch inflorescences in fields. Using weighted gene correlation network analysis (WGCNA), we identified several hub genes involved in the cytokinin metabolic pathway from modules highly associated with inflorescence phenotypes. Among them, *Jatropha ADENOSINE KINASE 2* (*JcADK2*), *ADENINE PHOSPHORIBOSYL TRANSFERASE 1* (*JcAPT1*), *CYTOKININ OXIDASE 3* (*JcCKX3*), *ISOPENTENYLTRANSFERASE 5* (*JcIPT5*), *LONELY GUY 3* (*JcLOG3*) and *JcLOG5* may participate in cytokinin metabolic pathway in *Jatropha*. Consistently, exogenous application of cytokinin (6-benzyladenine, 6-BA) on inflorescence buds induced high-branch inflorescence phenotype in both low-branch inflorescence mutant (*g*) and WT plants. These results suggested that cytokinin is an important regulator in controlling inflorescence branching in *Jatropha*. In addition, comparative transcriptome analysis showed that *Arabidopsis* homologous genes *Jatropha AGAMOUS-LIKE 6* (*JcAGL6*), *JcAGL24*, *FRUITFUL* (*JcFUL*), *LEAFY* (*JcLFY*), *SEPALLATAs* (*JcSEPs*), *TERMINAL FLOWER 1* (*JcTFL1*), and *WUSCHEL-RELATED HOMEOBOX 3* (*JcWOX3*), were differentially expressed in inflorescence buds between *dxh* and *g* mutants and WT plants, indicating that they may participate in inflorescence development in *Jatropha*. The expression of *JcTFL1* was downregulated, while the expression of *JcLFY* and *JcAP1* were upregulated in inflorescences in low-branch *g* mutant.

**Conclusions:**

Cytokinin is an important regulator in controlling inflorescence branching in *Jatropha.* The regulation of inflorescence architecture by the genes involved in floral development, including *TFL1*, *LFY* and *AP1*, may be conservative in *Jatropha* and *Arabidopsis*. Our results provide helpful information for elucidating the regulatory mechanism of inflorescence architecture in *Jatropha*.

## Background

Inflorescence architecture directly influencing plant reproductive success [1], is a key agronomic factor determining seed yield. In higher plants, inflorescence architectures exhibit remarkable diversity, which is derived from the inflorescence position on the shoot, the flower arrangement within inflorescence and the patterns and timing of flower initiation during reproductive development [2, 3]. Recently, a detailed review has been made on evolutionary developmental biology studies of inflorescence architecture [4]. In inflorescence development, the maintenance of the inflorescence meristem and initiation of the floral meristem are critical to determine inflorescence architecture. In *Arabidopsis thaliana*, CLAVATA (CLV) pathways regulate meristem maintenance by restricting the expression of *WUSCHEL* (*WUS*), which defines the stem cell niche [5, 6]. A mutation in *CLAVATA* (*CLV1*, *CLV2* or *CLV3*) results in the accumulation of meristem cells to generate an increased shoot meristem dome [7]. Mutation in *WUS* causes defective shoot and floral meristems [6]. In maize, *thick tassel dwarf1* (*td1*) and *fasciated ear2* (*fea2*) are orthologs of *CLV1* and *CLV2*, respectively. Loss-of-function mutant *td1* exhibits a fascinated ear with extra rows of kernels and a tassel with more dense spikelets [8]. The *fea2* mutant exhibits a massive ear inflorescence meristem and increased organ number [9].

In *Arabidopsis*, the transition time from the inflorescence to floral meristem is critical in the determination of inflorescence architecture; *TERMINAL FLOWER1* (*TFL1*), *LEAFY* (*LFY*) and *APETALA1* (*AP1*) participate in this process and regulate inflorescence branching patterns [10, 11]. *TFL* mutation promotes the conversion of the inflorescence meristem into the floral meristem, causing an abnormal inflorescence with a compound floral structure [12]. By contrast, the ectopic expression of *TFL1* in a transgenic plant induces a highly branched inflorescence phenotype in *Arabidopsis* [13]. Conversely, *LFY* and *AP1* repress the expression of *TFL1* in the floral meristem [11, 14]. Moreover, *SUPPRESSOR OF OVEREXPRESSION OF CONSTANS 1* (*SOC1*), *SHORT VEGETATIVE PHASE* (*SVP*), *AGAMOUS-LIKE 24* (*AGL24*), and *SEPALLATA 4* (*SEP4*) redundantly regulate inflorescence architecture by directly suppressing the expression of *TFL1*; a *soc1–2 agl24–3 svp-41 sep4–1* quadruple mutant displays a massive inflorescence branching phenotype [15].

Overexpression of rice *RCN1* and *RCN2* and maize *ZCN1*-*ZCN6*, which are homologous to *Arabidopsis TFL1*, causes highly branched inflorescences through maintaining the indeterminacy of the inflorescence meristem [16, 17]. Rice *TAWAWA1* (*TAW1*) regulates inflorescence architecture by suppressing the specification of the spikelet meristem to maintain the indeterminacy of inflorescence architecture [18]. A gain-of-function mutant, *tawawa1-D*, exhibits a highly branched inflorescence and increased number of spikelets, whereas a *TAW1* knockdown mutant displays a small, reduced branching inflorescence resulting from early termination of inflorescence meristems and the formation of spikelet meristems [18]. In rice, the *WEALTHY FARMER’S PANICLE* (*WFP*)/*IDEAL PLANT ARCHITECTURE* (*IPA1*) locus is linked to a *Squamosa Promoter Binding Protein-Like 14* (*OsSPL14*) gene that can be suppressed by OsmiR156 [19]. A single nucleotide change in *OsSPL14* that relieves the repression of OsmiR156 causes increased panicle branching and grain number [19, 20]. Overexpression of a maize *UNBRANCHED3* (*UB3*), an ortholog of rice *OsSPL14*, dramatically repressed tillering and panicle branching in rice; however, moderate expression of *UB3* slightly suppressed tillering, but promoted panicle branching, resulting in an increased grain number per panicle in rice [21, 22].

In maize, two *APETALA2*-like genes, *indeterminate spikelet1* (*ids1*) and *sister of indeterminate spikelet 1* (*SID1*), are required for inflorescence branching by regulating the initiation of spikelet meristem and floral meristem, and an *ids1 sid1* double mutant shows a reduced branching inflorescence phenotype [23]. Three maize genes, *RAMOSA1* (*RA1*), *RA2* and *RA3*, regulate the fate of the inflorescence meristem, and mutations in these genes cause an increased long-branching inflorescence [24–26].

Plant hormones such as auxin and cytokinin are required for the initiation and outgrowth of axillary meristems that generate inflorescence branches and florets during reproductive development [27, 28]. Several auxin biosynthesis and polar transport components, such as YUCCA (YUC), PIN-FORMED 1 (PIN1) and PINOID (PID), are important regulators in inflorescence development [27]. In *Arabidopsis*, the *YUC* gene encodes flavin monooxygenases (FMOs) that catalyze a rate-limiting step in tryptophan-dependent auxin biosynthesis; overexpression of *YUC* promotes auxin levels during development processes [29]. In maize, *sparse inflorescence1* (*spi1*), a homologous *Arabidopsis YUC*, encodes a FMO, and *spi1* mutation reveals defects in the initiation of axillary meristems and lateral organs, causing reduced branch number and floral organs in inflorescences [30]. Auxin transport proteins PIN1 and PID are required for the distribution of auxin; mutation in *pin1* or *pid* causes a pin-like inflorescence with abnormal flowers because of defects in the initiation of the axillary meristem in *Arabidopsis* [31, 32]. Maize *barreninflorescence2* (*bif2*), an ortholog of *PID*, is required for the initiation of the axillary meristem and lateral primordia; the *bif2* mutant displays a reduced number of branches, spikelets, florets and kernels in inflorescences [33]. *Barren inflorescence1* (*bif1*) mutation causes a similar phenotype to that of the *bif2* mutant [34]; the *barren stalk1* (*ba1*) mutant displays an unbranched inflorescence without spikelets because of a defect in auxin signaling [35]. In *Setaria viridis*, *SvAUXIN1* is required for inflorescence development and its loss-of-function mutant *sparse panicle1* (*spp1*) displays reduced and uneven inflorescence branching phenotype [36].

Cytokinin is required for meristem activity and plays a positive role in the shoot meristem [37]. In *Arabidopsis*, *CYTOKININ OXIDASE 3* (*CKX3*) and *CKX5* catalyze the degradation of cytokinin; *ckx3 ckx5* double mutant display larger inflorescences and floral meristems because of the accumulation of cytokinin [38]. In rice, a QTL locus, *Gn1*, encodes an OsCKX2; the reduced expression of *OsCKX2* causes higher cytokinin levels in the inflorescence meristem to increase the number of branches and spikelets, leading to enhanced grain yield [39]. A zinc finger transcription factor, DROUGHT AND SALT TOLERANCE (DST), directly regulates the accumulation of cytokinin in the shoot apical meristem (SAM) to promote panicle branching loading to the increase of grain number [40]. The *LONELY GUY* (*LOG*) gene encodes an enzyme that participates in the final step of bioactive cytokinin synthesis; mutation in *log* causes a small inflorescence with reduced branch and spikelet number [37].

At present, the studies on molecular mechanism of inflorescence architecture are focus on the model plants and few in perennial woody plants because of their long reproductive cycle and the difficulty in the establishment of genetic transformation system. To understand the regulatory mechanism of inflorescence architecture, the investigation of the evolutionary changes in developmental morphology and the identification of key factors controlling inflorescence development are crucial in closely related plant lineages that display different inflorescence structures [4], especially, in perennial woody plants.

*Jatropha curcas* L. has high seed oil content and is considered a potential biofuel plant [41, 42]. The *Jatropha* inflorescence exhibits a dichasial cyme pattern bearing male and female flowers in the same inflorescence. Few female flowers per inflorescence is considered one of the factors leading to poor seed yield in *Jatropha* [43]. Our previous research showed that co-suppression of *JcLFY* delayed flower formation, leading to production of more secondary inflorescence branches [44], but roles of *JcTFL1b* [44, 45] and *JcAP1* [46] in inflorescence branching remain unclear. Paclobutrazol (PAC), an inhibitor of gibberellin biosynthesis, causes compacted inflorescences with short branches, resulting in increased seed yield in *Jatropha* [47, 48]. Cytokinin treatment on inflorescence buds generated a larger inflorescence with a significantly increased the number of female and total flowers [43, 49, 50], which means a high-branch inflorescence phenotype. These results suggest that cytokinin may play a significant role in the determination of inflorescence architecture in *Jatropha*. In this study, two *Jatropha* mutants that exhibit different inflorescence branching traits were used for comparative transcriptome analysis to identify genes and regulation networks that participate in the regulation of inflorescence architecture. Our study will contribute to the understanding of the genetic basis of inflorescence architecture and to the breeding of high-yield *Jatropha* varieties.

## Results

### *dxh* and *g* mutants have different inflorescence branching phenotypes

**Fig. 1:**
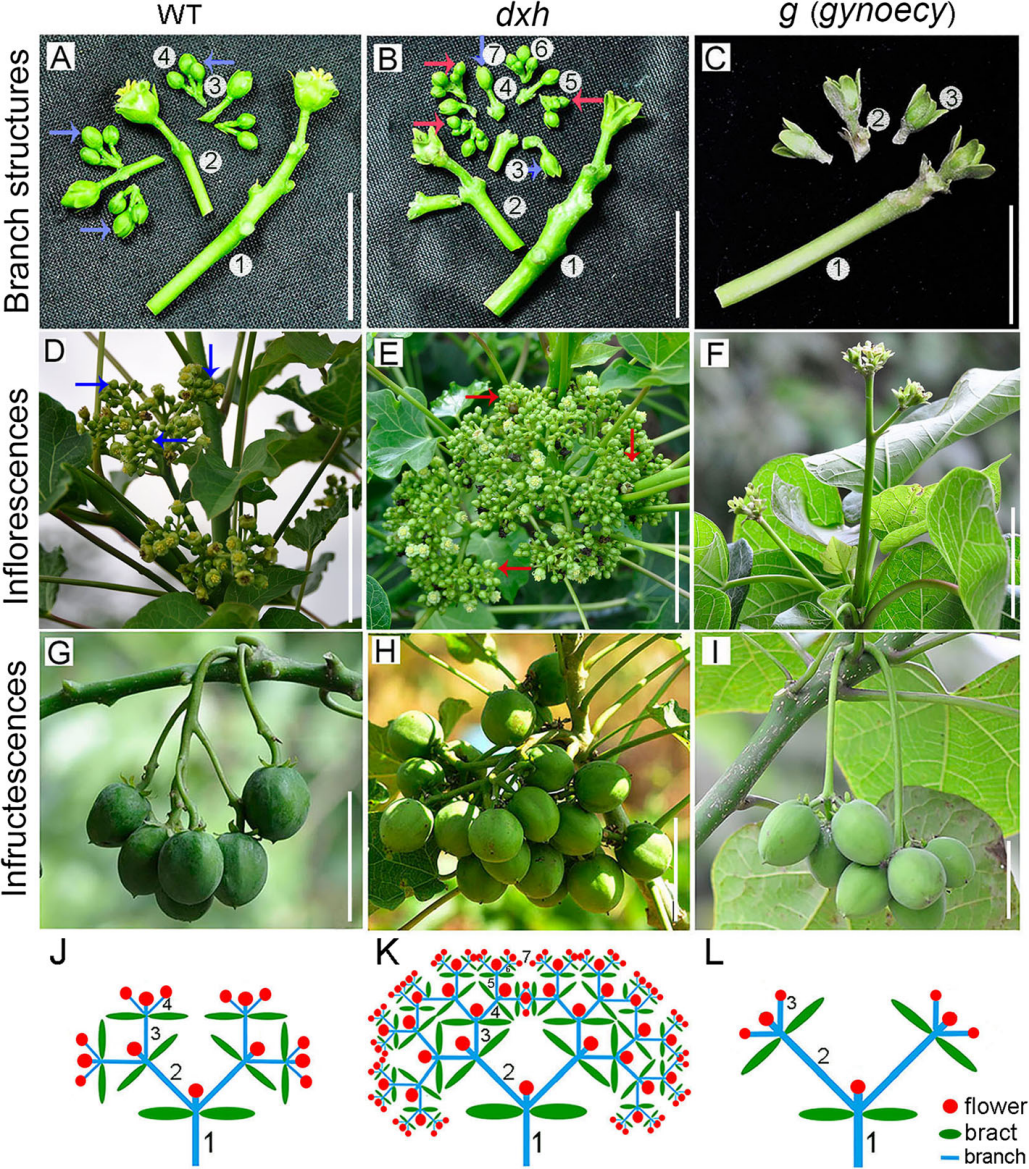
Phenotypes of inflorescence branching of WT and *dxh* and *g* mutants. **a**-**c** indicate the branching structure of inflorescences in WT and *dxh* and *g* mutants, respectively. Bar = 2.0 cm. **d**-**f** indicate the intact inflorescences of WT and *dxh* and *g* mutants. In panels **a**, **b**, **d** and **e**, the blue arrows indicate the fourth branching, and the red arrows denote the seventh branching. Bar = 5.0 cm. **g**-**i** indicate the infructescences of WT and *dxh* and *g* mutants. Bar = 5.0 cm. **j**-**l** indicate the diagrammatic sketch of inflorescence structure of WT and *dxh* and *g* mutants

**Fig. 2:**
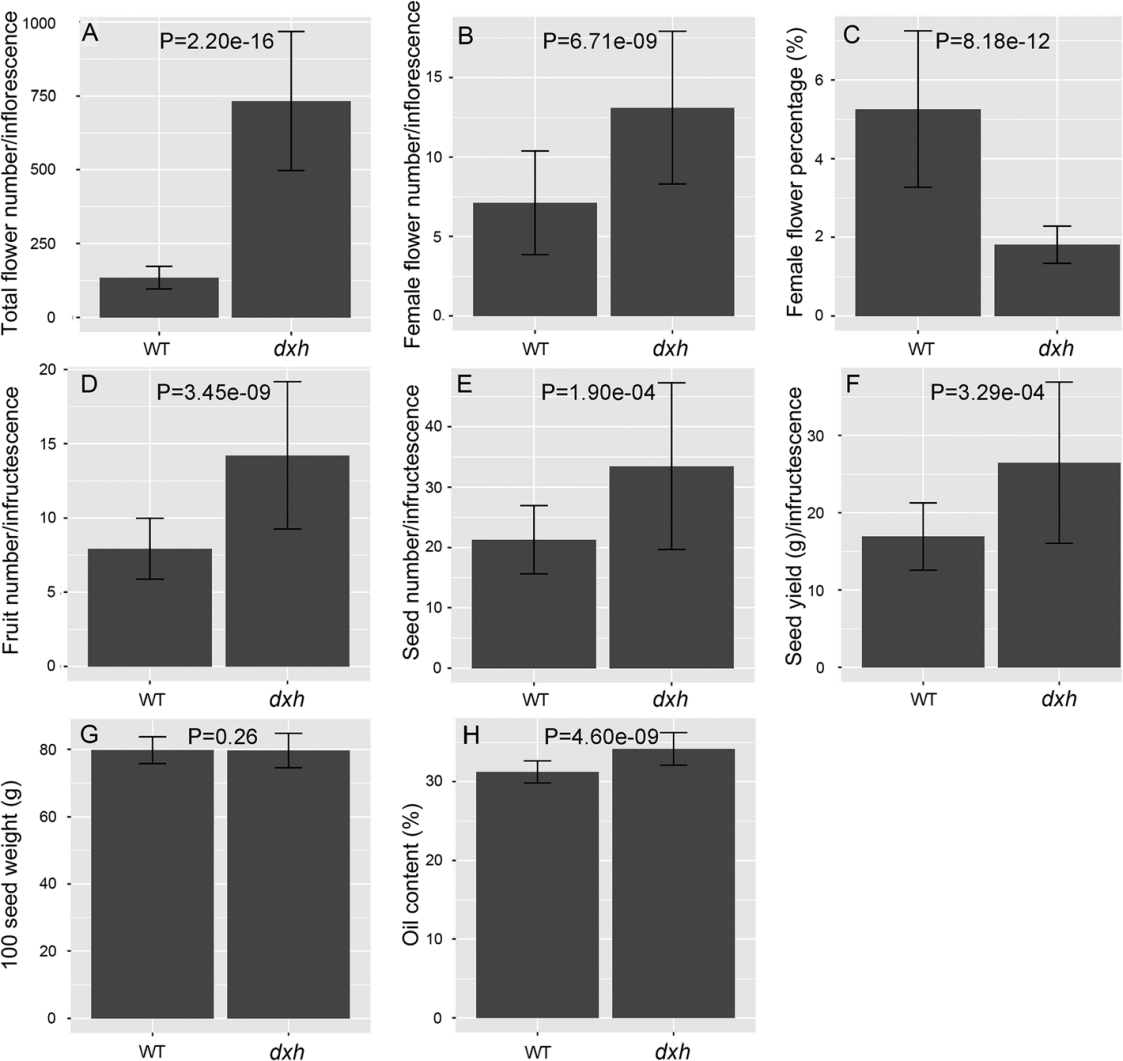
Comparison of agronomic traits between WT plants and *dxh* mutants. **a** Total flower number per inflorescence. **b** Female flower number per inflorescence. **c** Percentage of female flowers. **d** Fruit number per infructescence. **e** Seed number per infructescence. **f** Seed yield per infructescence. **g** 100-seed weight. **h** Seed oil content. The number of inflorescences/infructescences surveyed is 34–44; values are means ± SD for (**a**-**h**). Statistical test analysis was performed using the Welch two sample t-test in R software

In general, wild-type (WT) *Jatropha* has a four-order branch cyme inflorescence bearing female and male flowers, and three- or five-order branch inflorescence is occurred occasionally in different growth conditions. Here, we reported two inflorescence branching mutants: a high-branch *dxh* (Chinese for “more florets”) mutant with a seven-order branch inflorescence, which came from a mutagenized population treated with cobalt-60 gamma rays; a low-branch *g* mutant with a three-order branch inflorescence, which originated from a natural mutation [51] (Fig. 1). In the *dxh* mutant, the total flower number, female flower number, fruit number, seed number, seed yield and oil content were significantly increased, whereas the female-to-male ratio was decreased, and the 100-seed weight remained constant in *dxh* mutant plants compared with those in WT plants (Fig. 2). The increase in seed yield and oil content and the constant 100-seed weight in *dxh* plants indicated that the *dxh* mutant is an excellent material for breeding high-yield *Jatropha* varieties. The *g* mutant had a gynoecious genotype with normal female flowers, whereas male flowers were aborted, indicating that it is also good breeding material.

### Identification of differentially expressed genes

**Fig. 3:**
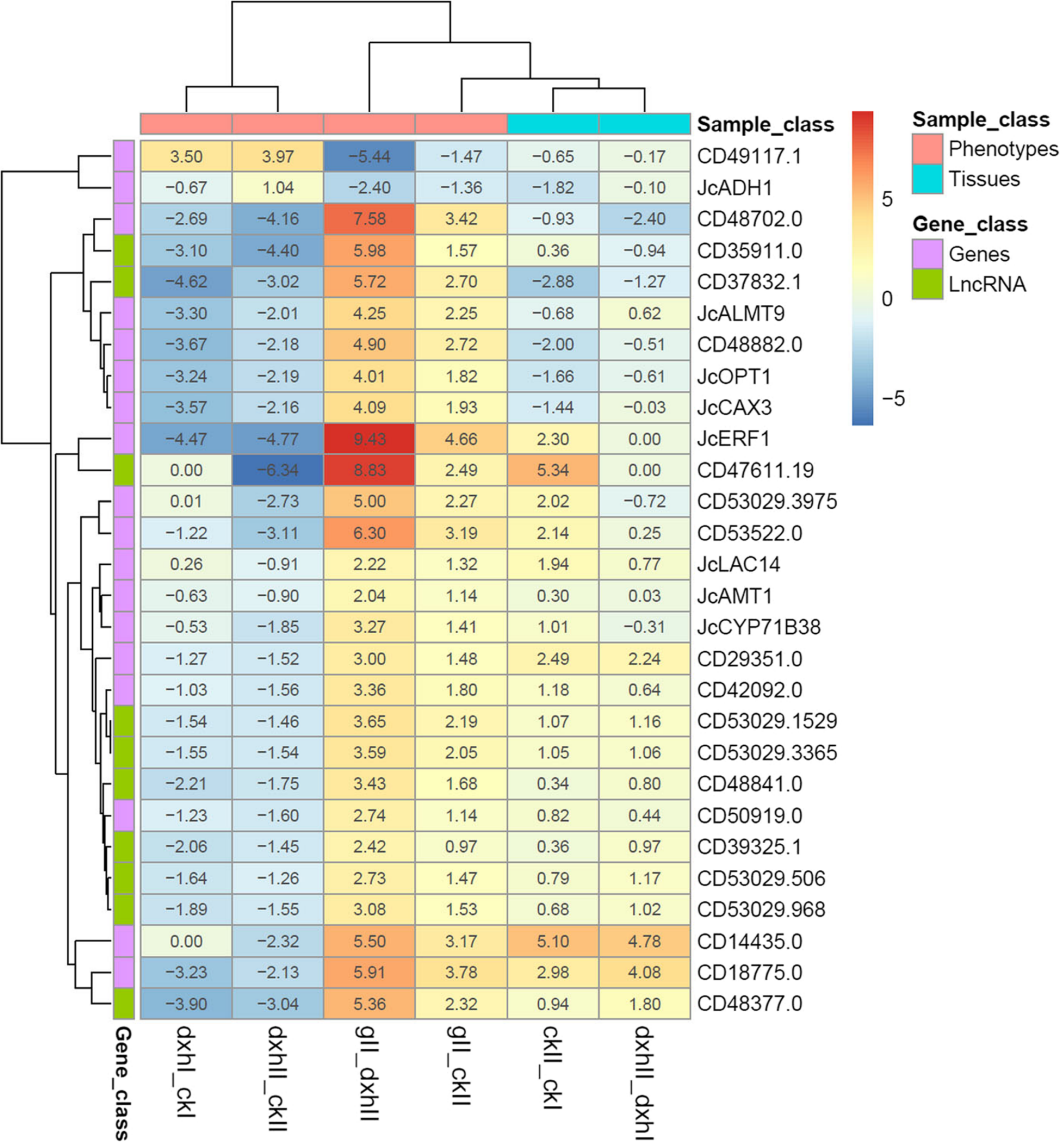
Hierarchical clustering of differentially expressed genes with opposite expression profiles in inflorescences between pairwise high-branch mutant vs. WT (dxhII_ckII) and low-branch mutant vs. WT (gII_ckII). The pairwise ckII_ckI and dxhII_dxhI indicate the comparisons of inflorescence bud and shoot tip in WT and *dxh* mutant, respectively; dxhI_ckI indicates the comparison of shoot tips between *dxh* mutant and WT; dxhII_ckII, gII_ckII and gII_dxhII indicate the comparisons of inflorescence buds between *dxh* and *g* mutant and WT, respectively. Genes shown in the figure is listed in Additional file 6. The color bar from red to blue indicates up-regulated and down-regulated expressions of genes in each pair; the dark color represents high fold change and the light color represents low fold change; the number in each color cell indicates corresponding value of fold change

To investigate the regulatory mechanism underlying inflorescence branching, we carried out RNA-Seq analysis between five group samples to identify DEGs (Additional file 1). These samples contained shoot tips that can generate inflorescence meristem and inflorescence buds from WT plants (hereafter referred to as ckI and ckII), shoot tips and inflorescence buds from the *dxh* mutant (referred to as dxhI and dxhII), and inflorescence buds from the *g* mutant (referred to as gII). A multidimensional scaling (MDS) plot showed that ckI, dxhI and gII samples can be well separated from ckII and dxhII based on the biological coefficient of variation (BCV) (Additional file 2). The close relationship between high-branch mutant (dxhII) and WT (ckII) inflorescence samples indicated that they have highly similar gene expression profiles. For simplicity, we hereafter refer to the comparison of ckII vs. ckI as ckII_ckI, dxhII vs. dxhI as dxhII_dxhI, dxhI vs. ckI as dxhI_ckI, dxhII vs. ckII as dxhII_ckII, gII vs. ckII as gII_ckII, and gII vs. dxhII as gII_dxhII. In the six pairs, 3549 DEGs in ckII_ckI, 410 in dxhII_dxhI, 2089 in dxhI_ckI, 329 in dxhII_ckII, 12,413 in gII_ckII and 12,282 in gII_dxhII were identified at an FDR of < 0.05 (Additional files 3 and 4). Among them, we identified 28 DEGs that have opposite expression patterns in inflorescences between pairwise high-branch mutant vs. WT (dxhII_ckII) and low-branch mutant vs. WT (gII_ckII) (Fig. 3 and Additional file 5). Expression of two genes of them were upregulated in inflorescences in pairwise high-branch mutant vs. WT (dxhII_ckII) and downregulated in pairwise low-branch mutant vs. WT (gII_ckII) while 26 genes were downregulated in pairwise gII_ckII, and upregulated in dxhII_ckII (Fig. 3). Interestingly, ten of them were long noncoding RNAs (LncRNAs) [52], suggesting that LncRNA may be involved in the regulation of inflorescence branching in *Jatropha*.

### Differentially expressed genes involved in inflorescence development

**Fig. 4:**
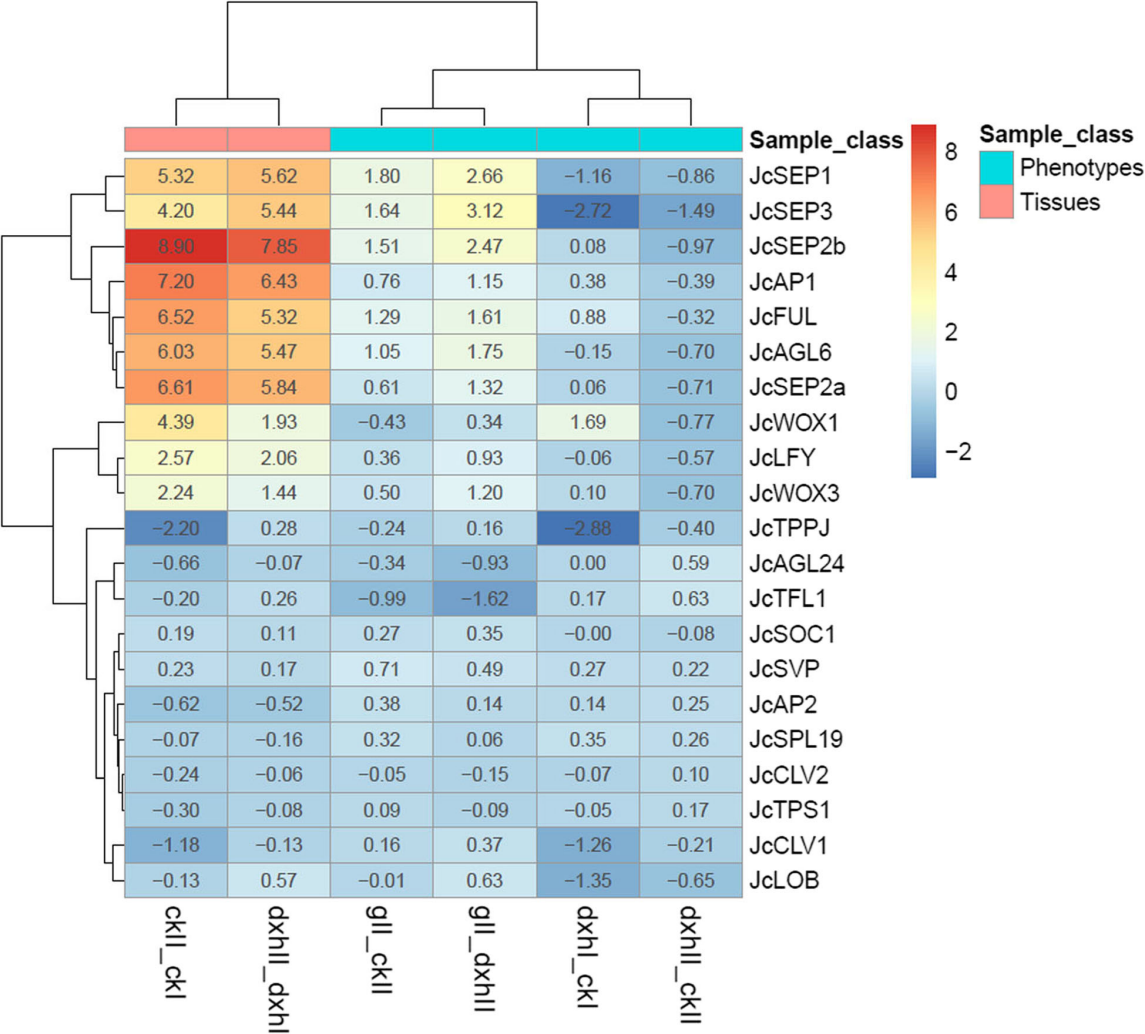
Hierarchical clustering of differentially expressed genes involved in inflorescence development. The pairwise ckII_ckI, dxhII_dxhI, dxhI_ckI, dxhII_ckII, gII_ckII and gII_dxhII indicate the same pairs shown in Fig. 3. The genes shown in the figure is listed in Additional file 7. The color bar from red to blue indicates up-regulated and down-regulated expressions of genes in each pair; the dark color represents high fold change and light color represents low fold change; the number in each color cell indicates corresponding value of fold change

According to *Arabidopsis* and rice genes involved in inflorescence development, we identified 21 homologous genes that may play similar roles in regulating *Jatropha* inflorescence development from our assembled data set. Nine of them were differentially expressed in inflorescences in pairwise low-branch vs. high-branch mutant (gII_dxhII) (Fold change ≥2.0 and FDR < 0.05) (Additional file 7). Most of them exhibited opposite expression patterns between pairwise high-branch mutant vs. WT (dxhII_ckII) and low-branch mutant vs. WT (gII_ckII) (Fig. 4), which are coincided with their respective phenotypes. Expression of *Jatropha AGAMOUS-LIKE 6* (*JcAGL6*), *FRUITFUL* (*JcFUL*), *JcLFY*, *SEPALLATA 1* (*JcSEP1*), *JcSEP2a*, *JcSEP2b*, *JcSEP3* and *WUSCHEL-RELATED HOMEOBOX 3* (*JcWOX3*) was upregulated, whereas expression of *JcAGL24* and *JcTFL1* was downregulated in pairwise low-branch vs. high-branch mutant (gII_dxhII). In *Arabidopsis*, *TFL1* promotes inflorescence branching, whereas *LFY* and *AP1* prevent inflorescence branching by repressing the expression of *TFL1* in the floral meristem [7, 8, 10]. Our results showed that *JcTFL1*, *JcLFY* and *JcAP1* may play similar roles in controlling inflorescence architecture in *Jatropha*, suggesting the function of these genes may be conservative in *Jatropha* and *Arabidopsis*.

### Differentially expressed genes involved in auxin and cytokinin metabolic and signaling pathways

**Fig. 5:**
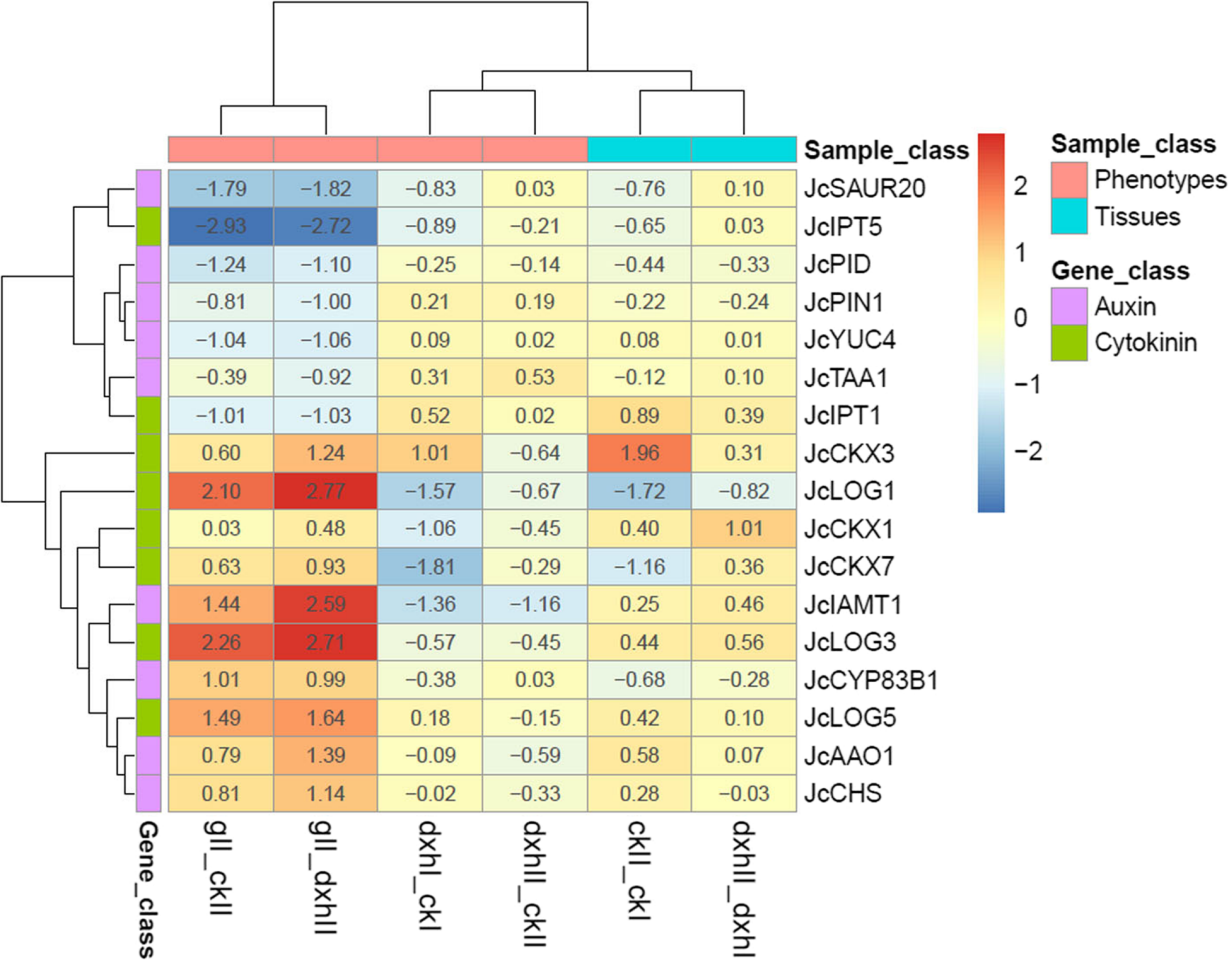
Hierarchical clustering of differentially expressed genes involved in auxin and cytokinin metabolic or signaling pathways. The pairwise ckII_ckI, dxhII_dxhI, dxhI_ckI, dxhII_ckII, gII_ckII and gII_dxhII indicate the same pairs shown in Fig. 3. The genes shown in the figure is listed in Additional file 8. The color bar from red to blue indicates up-regulated and down-regulated expressions of genes in every pair; the dark color represents high fold change and light color represents fold change; the number in each color cell indicates corresponding value of fold change

Auxin and cytokinin play important roles in inflorescence branching in *Arabidopsis* and rice [27, 28]. We identified 22 and 26 homologous genes involved in the auxin and cytokinin metabolic and signaling pathways, respectively, from our transcriptome data set (Additional file 8). Among them, *Jatropha ALDEHYDE OXIDASE 1* (*JcAAO1*), *CHALCONE SYNTHASE* (*JcCHS*), *cytochrome P450, family 83, subfamily B, polypeptide 1* (*JcCYP83B1*), *IAA carboxyl methyltransferase 1* (*JcIAMT1*), *JcPID*, *JcPIN1*, *SMALL AUXIN UP RNA 20* (*JcSAUR20*), and *YUCCA4* (*JcYUC4*) may be involved in the auxin metabolic and signaling pathways. *Jatropha CYTOKININ OXIDASE/DEHYDROGENASE 3* (*JcCKX3*), *JcCKX7*, *ISOPENTENYLTRANSFERASE 1* (*JcIPT1*), *JcIPT5*, *JcLOG1*, *JcLOG3*, and *JcLOG5* may be involved in the cytokinin metabolic and signaling pathways. All of them were differentially expressed in inflorescences in pairwise high-branch mutant vs. WT (dxhII_ckII) or low-branch mutant vs. WT (gII_ckII) (Fold change ≥2.0 and FDR < 0.05) (Fig. 5). Thus, the auxin and cytokinin metabolic or signaling pathways may participate in regulating inflorescence branching in *Jatropha*.

### Confirmation of expression profiles of candidate genes by real-time qPCR

To validate the result of transcriptome analysis, we selected a dozen candidate genes to test their expression patterns across the five group samples using the real-time qPCR method. These genes included *JcFUL*, *JcSEP1*, *JcSEP2A*, *JcSEP2B*, *JcSEP3*, *JcTFL1*, *JcCD35911.0*, *JcCD37832.1*, *JcCD39325.1*, *JcCD47611.19*, *JcCD53029.968* and *JcCD53029.1529* (Additional files 9 and 10). Correlation analysis showed that the expression patterns of these genes displayed by RNA-Seq were consistent with that by real-time qPCR (Additional file 11), indicating that the transcriptome results in this study are reliable.

### Construction of gene co-expression networks and identification of hub genes that regulate inflorescence branching

**Fig. 6:**
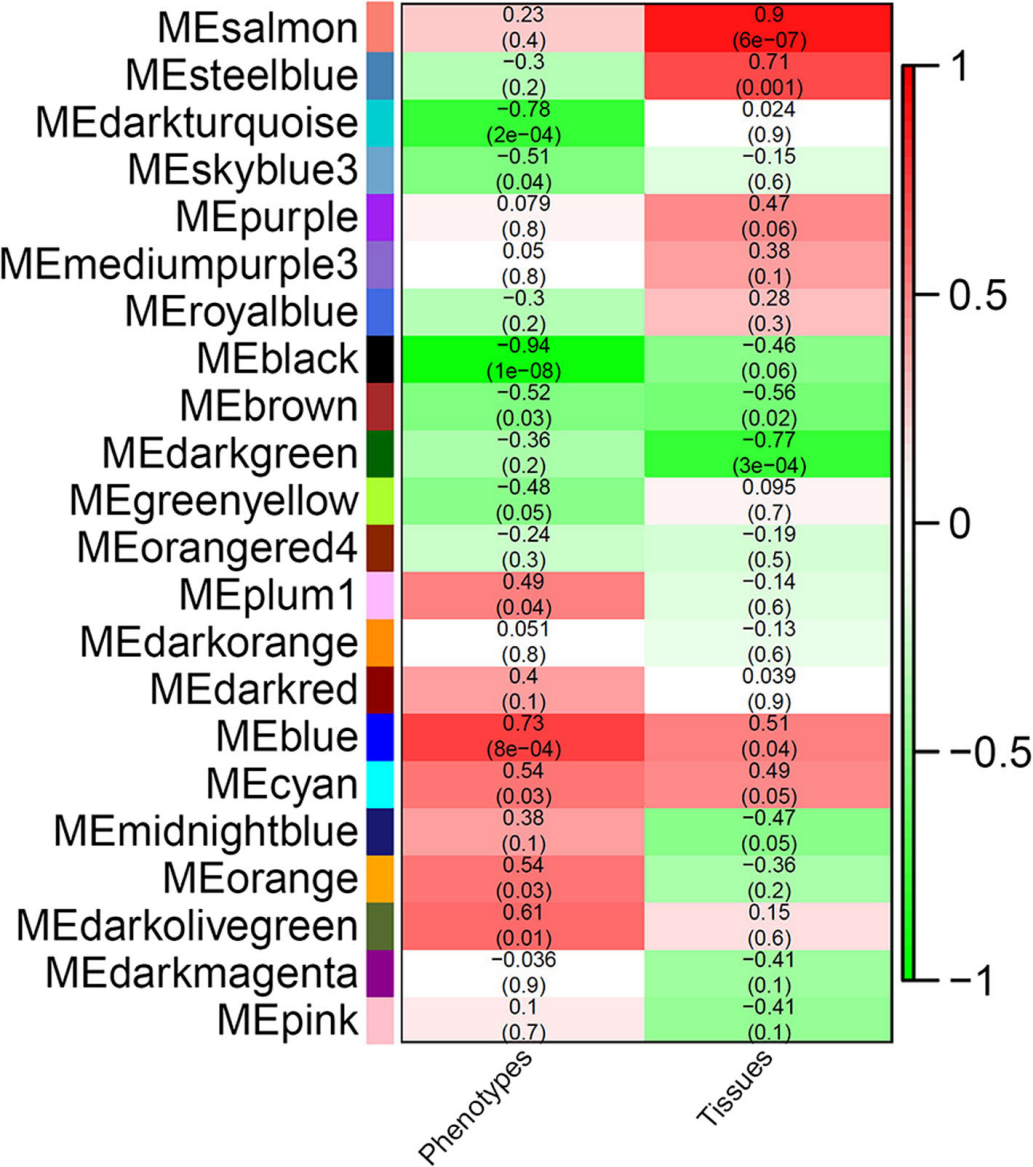
Correlation analysis between module eigengenes (ME) and biological traits (phenotypes and tissues) using weighted gene correlation network analysis (WGCNA). Each color block in left color column indicates a module identified (22 modules). The right color bar indicates the correlation coefficient; the red color represents the positive correlation and green represents the negative correlation. Two middle columns correspond to phenotype and tissues traits; the numbers in each color cell indicate the correlation coefficient and the corresponding *P*-value (numbers in brackets) between modules and two traits, calculated using the WGCNA package

**Fig. 7:**
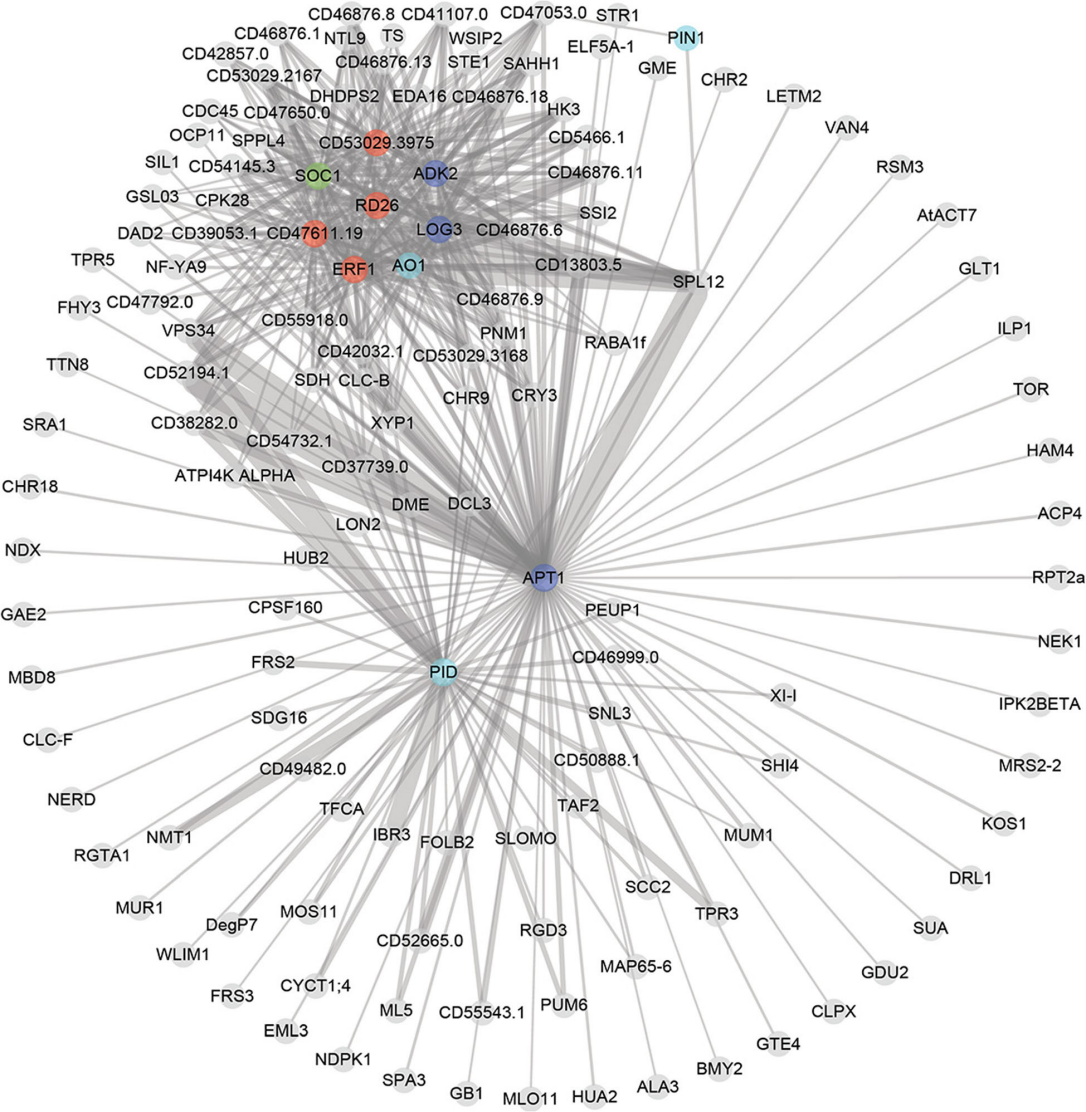
Co-expression network of genes in the MEblue module shown in Fig. 6. The red color indicates the differentially expressed genes with opposite expression profiles in inflorescences between pairwise high-branch mutant vs. WT (dxhII_ckII) and low-branch mutant vs. WT (gII_ckII) which are showed in Fig. 3; the green color indicates differentially expressed genes involved in inflorescence development which are showed in Fig. 4; the cyan color indicates differentially expressed genes in auxin metabolic and signaling pathways, and blue color indicates genes in cytokinin metabolic and signaling pathways which are showed in Fig. 5. Detailed information on genes shown in the figure is listed in Additional file 14

**Fig. 8:**
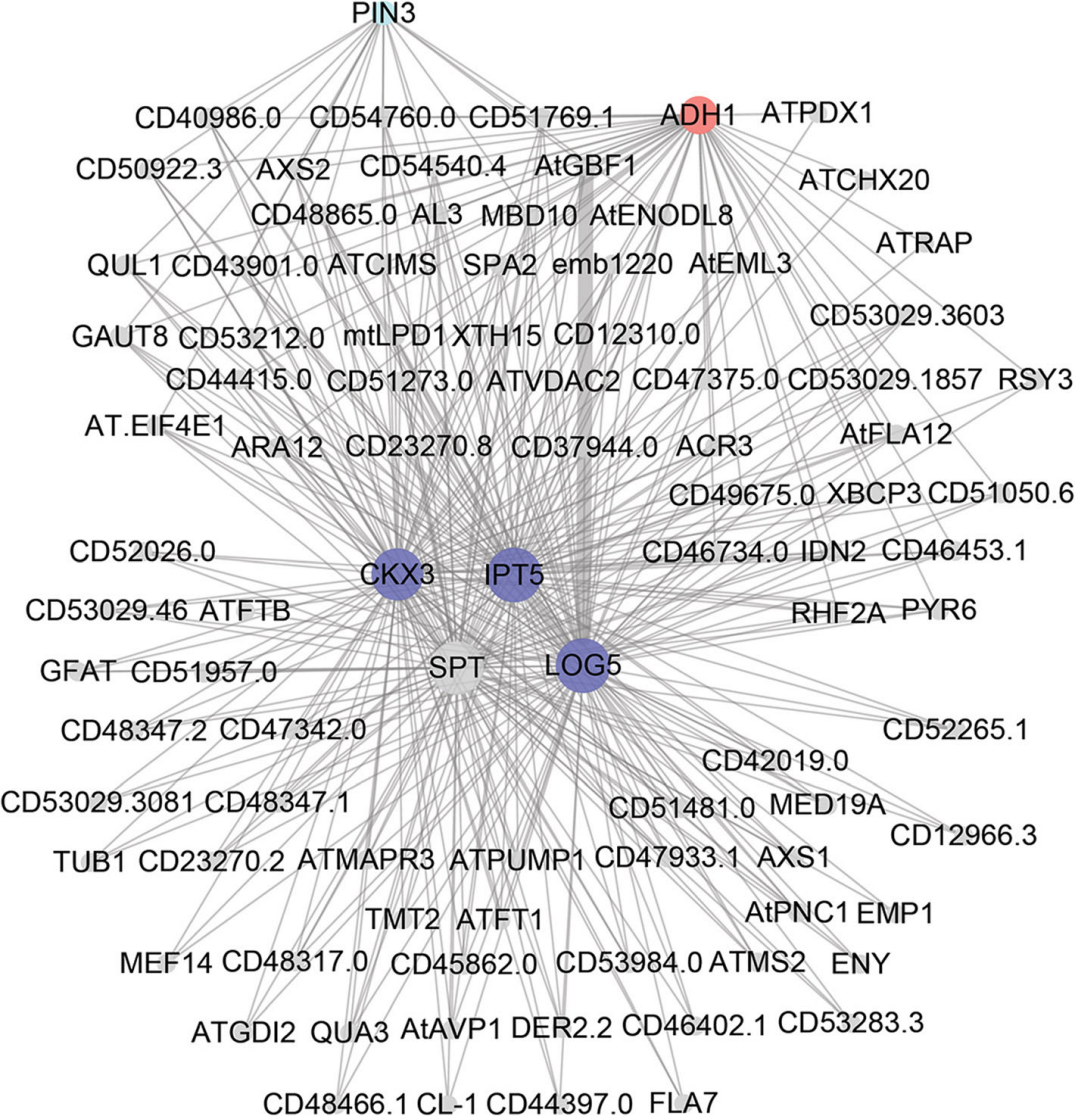
Co-expression network of genes in the MEblack module shown in Fig. 6. The red color indicates the differentially expressed genes with opposite expression profiles in inflorescences between pairwise high-branch mutant vs. WT (dxhII_ckII) and low-branch mutant vs. WT (gII_ckII) which are showed in Fig. 3; the cyan color indicates the differentially expressed genes in auxin metabolic and signaling pathways, and the blue color indicates cytokinin metabolic and signaling pathways which are showed in Fig. 5. Detailed information on the genes shown in the figure is listed in Additional file 14

We constructed a gene co-expression network using the WGCNA package [53]. In the network, 22 merged “modules” were identified; high correlation coefficients between genes in these modules indicated a high degree of interconnection (Additional file 12). The expression profile of each module was represented by its eigengene. We investigated the relationship between the module eigengenes and phenotype (three different inflorescence branching phenotypes) and tissue traits (shoot tips and inflorescence buds). MEblue and MEblack modules were highly associated with phenotype traits, and MEsalmon and MEdarkgreen modules were highly associated with deferent tissue traits (Fig. 6 and Additional file 13). We focused on the MEblue and MEblack modules because they might play more important roles in regulating inflorescence branching. According to the node connectivity that reflects how frequently a node interacts with others in a biological network and the results of transcriptome analysis, a dozen hub genes were identified from MEblue and MEblack modules (Figs. 7, 8 and Additional file 14). These genes include six *Arabidopsis* homologous genes, *Jatropha ADENOSINE KINASE 2* (*JcADK2*), *JcAPT1*, *JcCKX3*, *ISOPENTENYLTRANSFERASE 5* (*JcIPT5*), *LONELY GUY 3* (*JcLOG3*) and *JcLOG5* involved in the cytokinin metabolic pathway, four genes *JcAAO1*, *JcPID, JcPIN1,* and *JcPIN3* in the auxin biosynthetic and signaling pathways, *JcSOC1*, *SPATULA* (*JcSPT*) and five LncRNA genes. Among them, *JcADK2, JcAPT1*, *JcCKX3, JcIPT5, JcLOG3, JcLOG5, JcPID, JcPIN1* and *JcPIN3* were differentially expressed across different phenotypic inflorescences. The results showed that cytokinin and auxin metabolic or signaling pathways may participate in inflorescence development and may play vital roles in controlling inflorescence architecture.

### Application of 6-benzylaminopurine (6-BA) promoted an increase in inflorescence branches in both *g* mutant and WT plants

To validate the functions of cytokinin and auxin in inflorescence branching, we applied 6-BA (cytokinin) and 1-naphthaleneacetic acid (NAA, auxin) to the inflorescence buds of low-branch mutant (*g*) and WT plants, respectively. After 6-BA treatment, more than 70% of inflorescences displayed high-branch phenotype in both *g* mutant and WT plants, although the growth of some inflorescence buds was arrested (Additional file 15 A, E and C, G). A three-order branch inflorescence became a four-order branch inflorescence in *g* mutant plants (Additional file 15 B, F and A, E); and a five-order branch inflorescence became a six-order branch inflorescence in WT plants (Additional file 15 D, H and C, G), which is similar to the high-branch inflorescence phenotype of *dxh* mutant. We postulate that a strong cytokinin activity is present in *dxh* mutant inflorescence buds than in WT ones, which results in the high-branch inflorescence phenotype while a weak cytokinin activity in *g* mutant inflorescence buds leads to the low-branch inflorescence phenotype. However, NAA treatment had no effect on inflorescence branching (data not shown). These results indicate that cytokinin is an important regulator in regulating inflorescence branching in *Jatropha*.

## Discussion

In inflorescence development, cytokinin and auxin are essential for the formation and growth of the inflorescence branch meristem in *Arabidopsis* and rice [27, 28]. A gene co-expression network analysis showed that six homologous genes involved in the cytokinin metabolic pathway, and four genes in the auxin biosynthetic and signaling pathways are hub genes, which are from modules highly associated with inflorescence architecture phenotypes (Figs. 7 and 8), indicating that cytokinin and auxin likely to play important roles in regulating inflorescence branching in *Jatropha*. In cytokinin metabolic process, ADK catalyzes the phosphorylation of adenosine to AMP and converts cytokinin nucleosides to nucleotides contributing to intracellular CK homeostasis [54, 55]. ATP1 converts active cytokinin to inactive form and loss of ATP1 activity causes high accumulation of cytokinin bases evoking abnormal cytokinin-regulated responses [56]. Isopentenyltransferases (IPTs) catalyze the formation of isopentenyladenosine 5′-monophosphate (iPMP) from AMP and dimethylallylpyrophosphate (DMAPP), which is the first step of the cytokinin biosynthetic pathways [57]. LOG promotes cytokinin activity, and *log* mutants display a deficient in the maintenance of the shoot meristem in rice [28]. Cytokinin oxidases/dehydrogenases (CKXs) catalyze the irreversible degradation of cytokinins in cytokinin metabolic pathways [58]. In pairwise low-branch vs. high-branch mutant (gII_dxhII) and low-branch mutant vs. WT (gII_ckII) inflorescences, the expression of *JcIPT1* and *JcIPT5* was downregulated while the expression of *JcLOG1*, *JcLOG3*, *JcLOG5*, *JcCKX1*, *JcCKX3*, and *JcCKX7* was upregulated (Fig. 5). These results indicated that cytokinin biosynthesis is decreased, but cytokinin activation and degradation are promoted in low-branch mutant (*g*) inflorescence compared to those in high-branch mutant (*dxh*) and WT inflorescences. These effects may cause the low-branch inflorescence phenotype of *g* mutant.

In *Arabidopsis*, TRYPTOPHANAMINOTRANSFERASE OF ARABIDOPSIS 1 (TAA1) catalyzes the conversion from L-tryptophan (Trp) to indole-3-pyruvic acid (IPA), and YUC FMOs catalyze the oxidative decarboxylation of IPA to generate indole-3-acetic acid (IAA), both of which are the key enzymes in auxin biosynthesis [59, 60]. The expression of *JcTAA1* and *JcYUC4* was downregulated, indicating that auxin biosynthesis is decreased in low-branch (*g*) mutant inflorescence compared to that in high-branch (*dxh*) mutant inflorescence, along with the downregulated expression of *JcPIN1* and *JcPID* whose homologous genes serve as efflux carriers in auxin polar transport in *Arabidopsis* [61, 62] (Fig. 5). In addition, the expression of *JcSAUR20*, an auxin responsive gene, was downregulated, and *JcIAMT1* and *JcCHS* were upregulated, confirming that auxin biosynthesis may be decreased in low-branch mutant (*g*) inflorescence. In *Arabidopsis*, *IAMT1* encodes an IAA carboxyl methyltransferase that converts IAA to methyl-IAA ester (MeIAA); the overexpression of *MeIAA* causes dramatic hyponastic leaf phenotypes [63]. *CHS* encodes the first enzyme in flavonoid biosynthesis that is considered an auxin transport inhibitor [64]. However, there is no reasonable explanation for the upregulated expression of *JcAAO1* and *JcCYP83B1* genes, which are also involved in the auxin biosynthetic signaling pathways [65].

The application of 6-BA to inflorescence buds resulted in increased inflorescence branches both in low-branch mutant (*g*) and WT plants (Additional file 15). In our previous research, 6-BA treatment on WT inflorescence buds significantly promoted total flower number per inflorescence meaning a high-branch inflorescence phenotype, which is positive correlation to 6-BA concentration [49]. Thidiazuron (TDZ), another synthetic compound with cytokinin activity, was also shown to promote initial inflorescence branching in WT plants, although the final branch number will be decreased because of the abortion of flower buds [66]. These results supported that cytokinin is an important regulator in controlling inflorescence branching, which is in agreement with our results of WGCNA analysis. Mutation of several genes involved in the cytokinin metabolic or signaling pathways causes abnormal inflorescence branching phenotypes in *Arabidopsis* and rice [37–39], suggesting that cytokinin might has a conserved role in regulating inflorescence branching in different species.

## Conclusions

Using WGCNA, we identified several hub genes involved in the cytokinin metabolic pathway from modules highly associated with inflorescence architecture phenotypes. The application of cytokinin (6-BA) to inflorescence buds induced high-branch inflorescences both in low-branch mutant (*g*) and WT plants. These results supported that cytokinin is an important regulator and may play vital role in controlling inflorescence branching in *Jatropha.* Several *Arabidopsis* homologous genes involved in inflorescence development is significantly differentially expressed in inflorescence buds between mutants and WT plants, indicating that they participate in the regulation of inflorescence architecture in *Jatropha*. Based on the above results, we speculate that the change of inflorescence branching phenotype of two mutants may result from mutations at one or more loci in genome regions that contain genes involved in cytokinin metabolism and/or in inflorescence development. Our results will be helpful for elucidating the regulatory mechanism of inflorescence architecture in *Jatropha*.

## Methods

### Plant growth conditions and cytokinin (6-benzyladenine, 6-BA) treatment on inflorescence buds

The wild-type (WT), *duo xiao hua* (*dxh*) and *gynoecy* (*g*) mutant were grown in field in the Xishuangbanna Tropical Botanical Garden (XTBG) of the Chinese Academy of Sciences (21° N, 101° E) located in Mengla County, Yunnan Province, China. WT plant has a four-order branch inflorescence under normal growth conditions, occasionally a three- or five-order branch inflorescence. The *dxh* mutant has a high-branch inflorescence phenotype, a seven-order branch inflorescence, which was derived from a mutagenized population treated with cobalt-60 gamma rays. Its selfing progeny was selected until the stable high-branch inflorescence phenotype. *The g* mutant has a low-branch inflorescence phenotype, a three-order branch inflorescence, which was derived from a natural variation [51]. The cutting-propagated plants from single plant with stable phenotype were used for the preparation of experimental materials. Two-year-old cutting-propagated plants were grown in a field at 2 × 2 m per plant at the XTBG.

To confirm the effect of cytokinin on inflorescence branching, middle stage inflorescence buds (about 0.8 cm in diameter) that growth approximately 7–10 days from occurrence of invisible inflorescence bud of low-branch mutant (*g*) and WT plants were selected for once treatment with 1.0 mM 6-BA solution containing 0.05% Tween-20. The 6-BA and mock solutions were sprayed onto inflorescence buds wetting them to the point of run-off, ten inflorescence buds from three to five plants per treatment. After 2–3 weeks, inflorescence phenotypes were surveyed. Seed oil content was measured by using the minispec mq-one Seed Analyzer (Bruker Optik GmbH, Germany) as described previously [43], three replicates for each sample.

### Statistics of traits of high-branch inflorescence mutant (*dxh*) and WT

During the suitable period, the total flower number, female flower number, ratio of female-to-male flowers, fruit number, seed number, seed yield, weight of 100 seeds, and seed oil content per inflorescence/infructescence were surveyed in high-branch inflorescence mutant (*dxh*) and WT, respectively. In total 33 inflorescence/infructescence are surveyed in *dxh* and 44 ones in WT. Statistical test analysis was performed using the Welch two sample t-test in R software (https://cran.r-project.org).

### Collection of samples, RNA isolation and library construction

At initial reproductive period, after removed leaves, shoot tips that can generate inflorescence meristem were harvested from *dxh* and WT plants. Inflorescence buds that growth approximately 3–4 days from occurrence of invisible inflorescence bud (about 0.4 cm in diameter) were harvested from *dxh* and *g* mutants and WT plants. Three shoot tips or inflorescence buds were pooled as one biological replicate for RNA isolation, three replicates per sample. Total RNA extraction, library construction and quality control were performed as previously described [51]. Sequencing was performed on an Illumina Hiseq 2500 platform by Novogene Bioinformatics Technology (Beijing, China).

### De novo transcriptome assembly and read mapping

Raw reads were treated with the Fastq_clean [67] and assessed with FASTQC (http://www.bioinformatics.babraham.ac.uk/projects/fastqc). De novo transcriptome assembly was performed using Trinity (version 2.0.6) with default parameters [68, 69]. In all 122,526 sequences were generated. Bowtie version 1.1.1 (−v 2 -m 10) was used for the mapping of the paired-end reads from each library [70].

### Identification of differentially expressed transcripts

The Corset (version 1.03) was used for abundance estimation of transcripts [71]. Differentially expressed transcripts (DEGs) with a false discovery rate (FDR) of < 0.05 were identified by using the edgeR package [72]. The Venny (version 2.1) was used for the generation of venn diagram of DEGs (http://bioinfogp.cnb.csic.es/tools/venny/index.html). Hierarchical clustering of transcripts was performed using the pheatmap R package (version 1.0.7) (https://github.com/cran/pheatmap).

### Annotation of transcripts

A total of 16,206 filtered transcripts used for differentially expressed analysis were annotated with BLASTX search against the Ensembl Plants database (http://plants.ensembl.org) with Evalue < 1.0E-05. Among them, 14,680 transcripts were annotated and 1526 were not found (Additional file 16). Ten transcripts displayed in Fig. 3 were defined as LncRNAs because they have not annotation, not coding protein and length > 200 bp [52].

### Validation of expression profiles of candidate genes by real-time PCR (qPCR)

RNA samples for qPCR are same as RNA-seq ones. The cDNA was synthesized from total RNA (1.0 μg) using a PrimeScript RT Reagent Kit (Takara, Otsu, Japan), for each sample. qPCR was performed on a LightCycler 480 II (Roche, Penzberg, Germany) using the SYBR green I Kit (Roche), with three independent biological replicates for each sample and three technical replicates. *JcGAPDH* was as the internal reference. Primers for qPCR were listed in Additional file 10. The relative expression levels of genes were calculated by the 2^−ΔΔ^ CT method. Correlation analysis between RNA-Seq and qPCR expression data of the genes is performed with cor.test in R software (https://cran.r-project.org).

### Construction and analysis of weighted gene co-expression networks

The raw count data of differentially expressed genes, which were transformed with Log_2_(x + 1), from edgeR were applied to construct co-expression networks using the R package weighted gene correlation network analysis (WGCNA) [53]. The soft thresholding power of 6 was chosen based on the criterion of approximate scale-free topology. The minimum module size was 30, and modules were merged with the cutoff value of 0.2. The interaction network was visualized using the Cytoscape software [73].

## Supplementary information

**Additional file 1.** Sequencing read counts, quality, and alignment statistics for 15 *Jatropha* samples.

**Additional file 2.** Relationships of 15 inflorescence bud samples based on multidimensional scaling (MDS) analysis. The MDS plot is generated using plotMDS function in edgeR package. The distances between samples correspond to biological coefficient of variation (BCV) between those samples. ckI indicates the shoot tips of WT, containing ckI_1, ckI_2 and ckI_3 samples; ckII indicates the inflorescence buds of WT, containing ckII_1, ckII_2 and ckII_3 samples; dxhI indicates the shoot tips of *dxh* mutant, containing dxhI_1, dxhI_2 and dxhI_3 samples; dxhII indicates the inflorescence buds of *dxh* mutant, containing dxhII_1, dxhII_2 and dxhII_3 samples; and gII indicates the inflorescence buds of *g* mutant, containing gII_1, gII_2 and gII_3 samples, respectively.

**Additional file 3.** Differentially expressed genes in inflorescences between six pairs in *Jatropha*. The pairwise ckII_ckI, dxhII_dxhI, dxhI_ckI, dxhII_ckII, gII_ckII and gII_dxhII indicate the same pairs shown in Fig. 3; blue lines indicate genes with a two-fold change; red points indicate genes with significantly different expression at a false discovery rate (FDR) of < 0.05. FC, fold change; CPM, counts per million mapped reads.

**Additional file 4.** List of differentially expressed genes identified in inflorescences between six pairs in *Jatropha*. The pairwise ckII_ckI, dxhII_dxhI, dxhI_ckI, dxhII_ckII, gII_ckII and gII_dxhII indicate the same pairs shown in Fig. 3.

**Additional file 5.** The overlap of differentially expressed genes in inflorescences between six pairs in *Jatropha*. The pairwise ckII_ckI, dxhII_dxhI, dxhI_ckI, dxhII_ckII, gII_ckII and gII_dxhII indicate the same pairs shown in Fig. 3. All differentially expressed transcripts in Additional file 5 were listed in Additional file 4.

**Additional file 6.** Differentially expressed genes with opposite expression patterns in inflorescences between pairwise dxhII_ckII and gII_dxhII.

**Additional file 7.** Homologous genes involved in inflorescence development in *Jatropha*.

**Additional file 8.** Homologous genes involved in the auxin and cytokinin metabolic and signaling pathways in *Jatropha*.

**Additional file 9.** Validation of the expression profiles of 12 candidate genes by real-time qPCR. ckI and dxhI indicate the shoot tips of WT plants and *dxh* mutants; ckII, dxhII and gII indicate inflorescence buds of WT, *dxh* and *g* mutants, respectively. *JcGAPDH* was as the internal reference. The error bars represent SD (*n* = 3).

**Additional file 10.** List of primers for qPCR validation.

**Additional file 11.** Correlation analysis between RNA-Seq and qPCR expression data of the genes shown in Additional file 9. The correlation analysis is performed with cor.test in R software. FC, fold change.

**Additional file 12.** Weighted co-expression network and modules identified by WGCNA analysis. Each leaf in the hierarchical cluster tree represents one gene; each row and column of the heat map plot corresponds to one gene; in the heat map, light color indicate weak co-expression, and dark color indicates strong co-expression; twenty-two modules were labeled by different colors.

**Additional file 13.** Correlation analysis of modules and phenotype traits. Each row and column represent one module; the red color represents high adjacency (positive correlation), and blue color represents low adjacency (negative correlation); red squares along the diagonal indicate the modules with similar expression patterns.

**Additional file 14.** Gene list in MEblack and MEblue modules.

**Additional file 15.** Application of 6-benzylaminopurine (6-BA) promotes inflorescence branching of *g* mutants and WT plants. (A)-(D) show the complete inflorescence, and (E)-(H) show the dissected inflorescence, respectively. (A) and (E) indicate an increased inflorescence branching treated with 6-BA and (B) and (F) display a normal inflorescence branching of *g* mutants with mock. (C) and (G) indicate an increase inflorescence branching treated with 6-BA and (D) and (H) display a normal inflorescence branching of WT plants with mock. The numbers in (E)-(H) represent different orders of inflorescence branching. Bar = 5.0 cm.

**Additional file 16.** Annotation of transcripts.

**Additional file 17.** Transcripts sequences used for the differential expression analysis.

## Data Availability

RNA-Seq data from *dxh* and WT samples were deposited in NCBI under the accession number SRP122257. RNA-Seq data from three *g* samples were deposited under accession numbers SRR4473569, SRR4473570 and SRR4473575 [51]. Transcriptome sequences referred to the differential expression analysis of genes were listed in Additional file 17.

## Abbreviations

6-BA: 6-Benzyladenine
AAO: Aldehyde oxidase
ADK: Adenosine kinase
AGL: Agamous-like
AMP: Activated protein kinase
AP1: Apetala1
APT: Adenine phosphoribosyl transferase
ATP: Atairp2 target protein
BA1: Barren stalk1
BIF: Barren infloresccence
CHS: Chalcone synthase
CKX: Cytokinin oxidase/dehydrogenase
CLV: Clavata
CPM: Counts per million mapped reads
CR: Cox-reid profile-adjusted likelihood
CYP: Cytochrome P450
DEG: Differentially expressed genes
DMAPP: Dimethylallylpyrophosphate
DST: Drought and salt tolerance
DXH: Duo xiao hua
FC: Fold change
FDR: False discovery rate
FEA2: Fasciated ear2
FMO: Flavin monooxygenase
FUL: Fruitful
G: Gynoecy
GLM: Generalized linear model
IAA: Indol-3-acetic acid
IAMT: IAA carboxylmethyltransferase
IDS: Indeterminate spikelet
IPA: Indole-3-pyruvic acid
iPMP: Isopentenyladenosine 5′-monophosphate
IPT: Isopentenyltransferase
LFY: Leafy
LncRNA: Long noncoding RNA
LOG: Lonely guy
MDS: Multidimensional scaling
MeIAA: Methyl-IAA ester
MIR56: Microrna156
NAA: 1-Naphthaleneacetic acid
PID: Pinoid
PIN: Pin-formed
qPCR: Real-time quantitative PCR
QTL: Quantitative trait locus
RA: Ramosa
RCN: Rice terminal flower 1/centroradialis homologs
RNA-Seq: RNA-sequence
SAM: Shoot apical meristem
SAUR: Small auxin up RNA
SEP: Sepallata
SID: S ister of indeterminate spikelet
SOC1: Suppressor of overexpression of constans 1
SPI1: Sparse inflorescence1
SPL: Squamosa promoter binding protein-like
SPT: Spatula
SVP: Short vegetative phase
TAA: Tryptophanaminotransferase of *Arabidopsis*
TAW1: Tawawa1
TD1: Tassel dwarf1
TDZ: Thidiazuron
TFL1: Terminal flower1
TRP: Tryptophan
UB3: Unbranched3
WFP: Wealthy farmer’s panicle
WGCNA: Weighted gene correlation network analysis
WOX: Wuschel-related homeobox
WT: Wild-type
WUS: Wuschel
YUC: Yucca
ZCN: Zea centroradialis
