# Supplementary material for: Transcriptome analysis of two inflorescence branching mutants reveals cytokinin is an important regulator in controlling inflorescence architecture in the woody plant Jatropha curcas
Source: BMC Plant Biol. 2019 Nov 4;19:468. doi: 10.1186/s12870-019-2069-3 (PMC6830001; doi:10.1186/s12870-019-2069-3)
Supplement: Supplementary file 1 — Additional file 1. Sequencing read counts, quality, and alignment statistics for 15 Jatropha samples. [file 12870_2019_2069_MOESM1_ESM.docx]

Additional file 1 Sequencing read counts, quality, and alignment statistics for 15 *Jatropha* samples

| **ID** | **Clean Pairs** | **Error (%)** | **Q30 (%)** | **Mapped (%)** | **Sample name** |
| --- | --- | --- | --- | --- | --- |
| ckI_1_1 | 27254450 | 0.03 | 89.06 | 86.07 | Wild inflorescence  at stage I (ckI) |
| ckI_1_2 | 27254450 | 0.04 | 87.89 |  |  |
| ckI_2_1 | 26025031 | 0.03 | 93.37 | 85.97 |  |
| ckI_2_2 | 26025031 | 0.04 | 88.31 |  |  |
| ckI_3_1 | 28561841 | 0.03 | 92.89 | 85.49 |  |
| ckI_3_2 | 28561841 | 0.04 | 89.60 |  |  |
| ckII_1_1 | 22739342 | 0.03 | 93.20 | 85.88 | Wild inflorescence  at stage II (ckII) |
| ckII_1_2 | 22939342 | 0.04 | 88.37 |  |  |
| ckII_2_1 | 24992042 | 0.03 | 93.09 | 86.23 |  |
| ckII_2_2 | 24121065 | 0.04 | 87.77 |  |  |
| ckII_3_1 | 24121065 | 0.03 | 93.27 | 85.75 |  |
| ckII_3_2 | 26144798 | 0.04 | 87.85 |  |  |
| dxhI_1_1 | 21777979 | 0.03 | 93.87 | 87.37 | *dxh* inflorescence  at stage I (dxhI) |
| dxhI_1_2 | 21777979 | 0.04 | 89.18 |  |  |
| dxhI_2_1 | 24409372 | 0.03 | 93.88 | 86.26 |  |
| dxhI_2_2 | 24409372 | 0.04 | 89.26 |  |  |
| dxhI_3_1 | 20674839 | 0.03 | 93.88 | 86.23 |  |
| dxhI_3_2 | 20674839 | 0.04 | 89.10 |  |  |
| dxhII_1_1 | 25315410 | 0.03 | 94.02 | 87.43 | *dxh* inflorescence  at stage II (dxhII) |
| dxhII_1_2 | 25315410 | 0.04 | 89.40 |  |  |
| dxhII_2_1 | 23234366 | 0.03 | 93.92 | 86.75 |  |
| dxhII_2_2 | 23234366 | 0.04 | 89.45 |  |  |
| dxhII_3_1 | 23717960 | 0.03 | 93.80 | 87.64 |  |
| dxhII_3_2 | 23717960 | 0.04 | 89.06 |  |  |
| gII_1_1 | 25925411 | 0.03 | 93.17 | 85.70 | *g* inflorescence  at stage II (gII) |
| gII_1_2 | 25925411 | 0.04 | 89.17 |  |  |
| gII_2_1 | 24357828 | 0.03 | 92.78 | 88.43 |  |
| gII_2_2 | 24357828 | 0.03 | 90.97 |  |  |
| gII_3_1 | 31025373 | 0.03 | 93.15 | 85.06 |  |
| gII_3_2 | 31025373 | 0.04 | 89.65 |  |  |

Mapped read indicates the paired reads with at least one reported alignment.
