# Supplementary figures and images for: Transcriptome analysis of two inflorescence branching mutants reveals cytokinin is an important regulator in controlling inflorescence architecture in the woody plant Jatropha curcas
Source: BMC Plant Biol. 2019 Nov 4;19:468. doi: 10.1186/s12870-019-2069-3 (PMC6830001; doi:10.1186/s12870-019-2069-3)

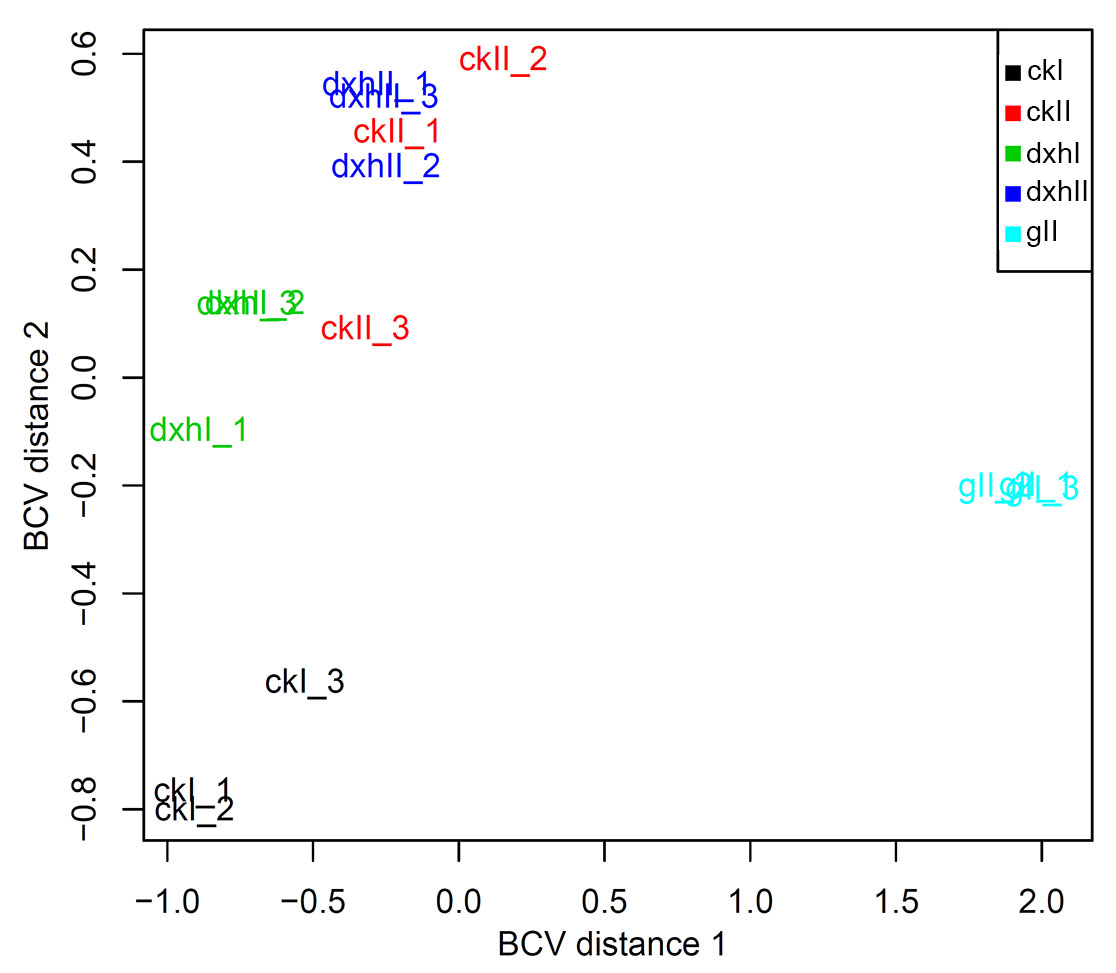

Supplement: Supplementary file 2 — Additional file 2. Relationships of 15 inflorescence bud samples based on multidimensional scaling (MDS) analysis. The MDS plot is generated using plotMDS function in edgeR package. The distances between samples correspond to biological coefficient of variation (BCV) between those samples. ckI indicates the shoot tips of WT, containing ckI_1, ckI_2 and ckI_3 samples; ckII indicates the inflorescence buds of WT, containing ckII_1, ckII_2 and ckII_3 samples; dxhI indicates the shoot tips of dxh mutant, containing dxhI_1, dxhI_2 and dxhI_3 samples; dxhII indicates the inflorescence buds of dxh mutant, containing dxhII_1, dxhII_2 and dxhII_3 samples; and gII indicates the inflorescence buds of g mutant, containing gII_1, gII_2 and gII_3 samples, respectively. [file 12870_2019_2069_MOESM2_ESM.jpg]

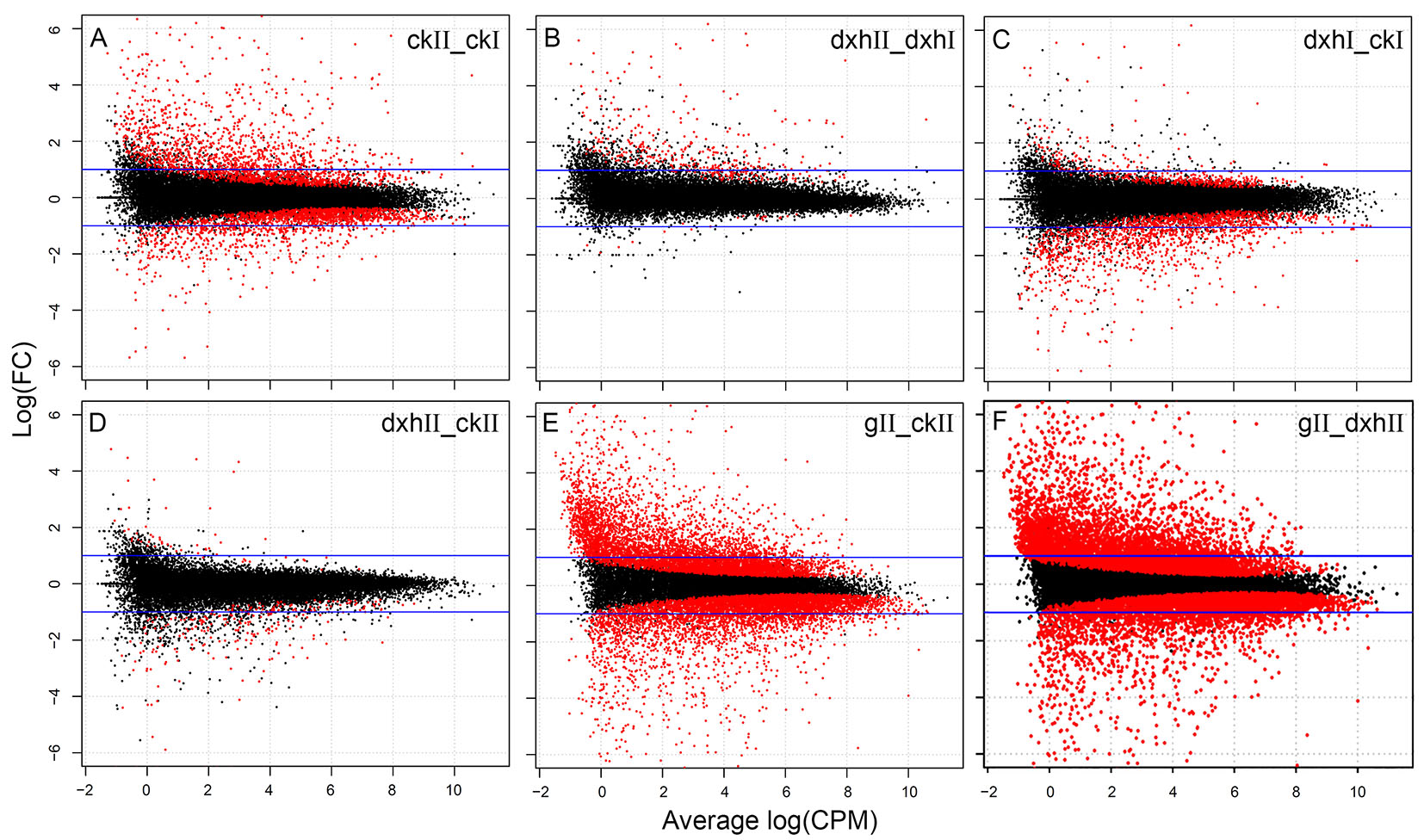

Supplement: Supplementary file 3 — Additional file 3. Differentially expressed genes in inflorescences between six pairs in Jatropha. The pairwise ckII_ckI, dxhII_dxhI, dxhI_ckI, dxhII_ckII, gII_ckII and gII_dxhII indicate the same pairs shown in Fig. 3; blue lines indicate genes with a two-fold change; red points indicate genes with significantly different expression at a false discovery rate (FDR) of < 0.05. FC, fold change; CPM, counts per million mapped reads. [file 12870_2019_2069_MOESM3_ESM.jpg]

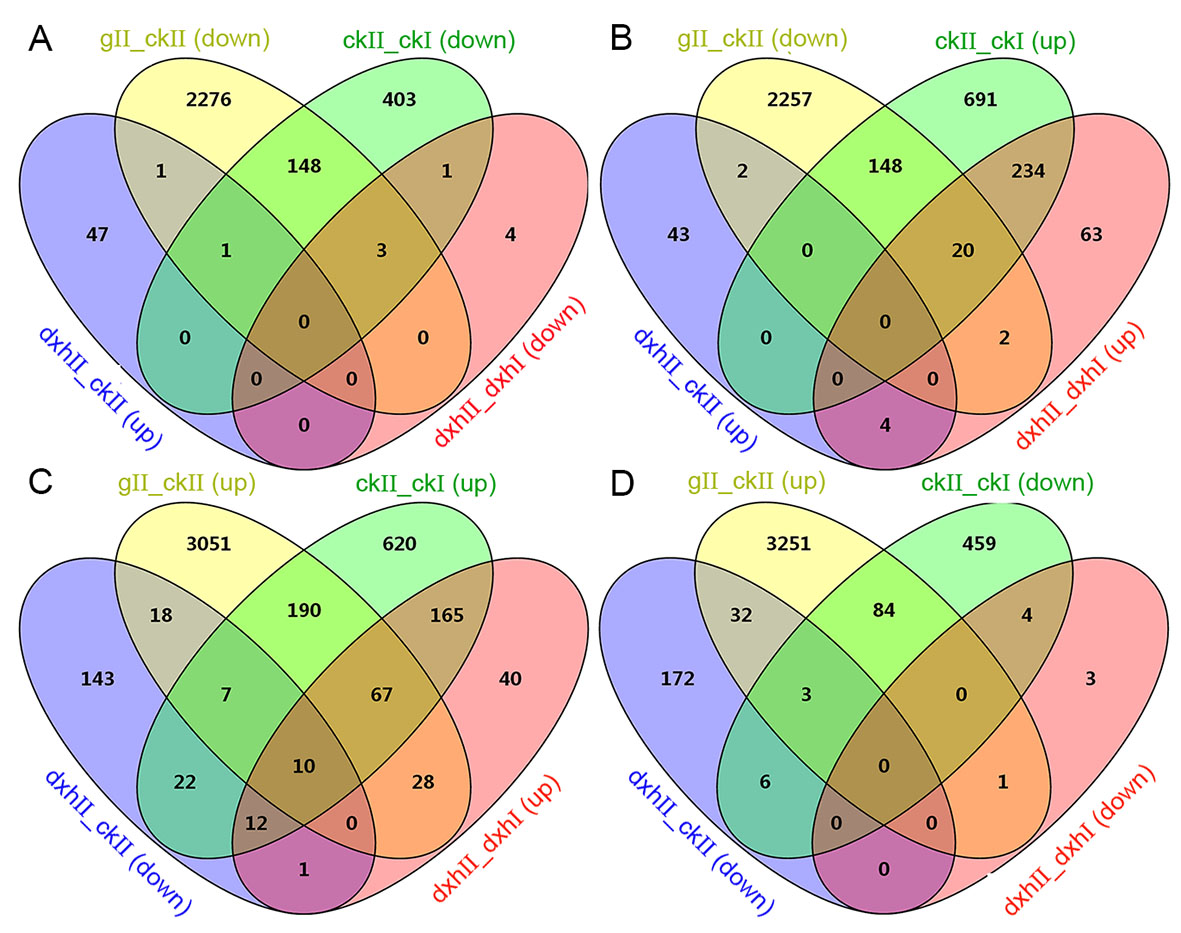

Supplement: Supplementary file 5 — Additional file 5. The overlap of differentially expressed genes in inflorescences between six pairs in Jatropha. The pairwise ckII_ckI, dxhII_dxhI, dxhI_ckI, dxhII_ckII, gII_ckII and gII_dxhII indicate the same pairs shown in Fig. 3. All differentially expressed transcripts in Additional file 5 were listed in Additional file 4. [file 12870_2019_2069_MOESM5_ESM.jpg]

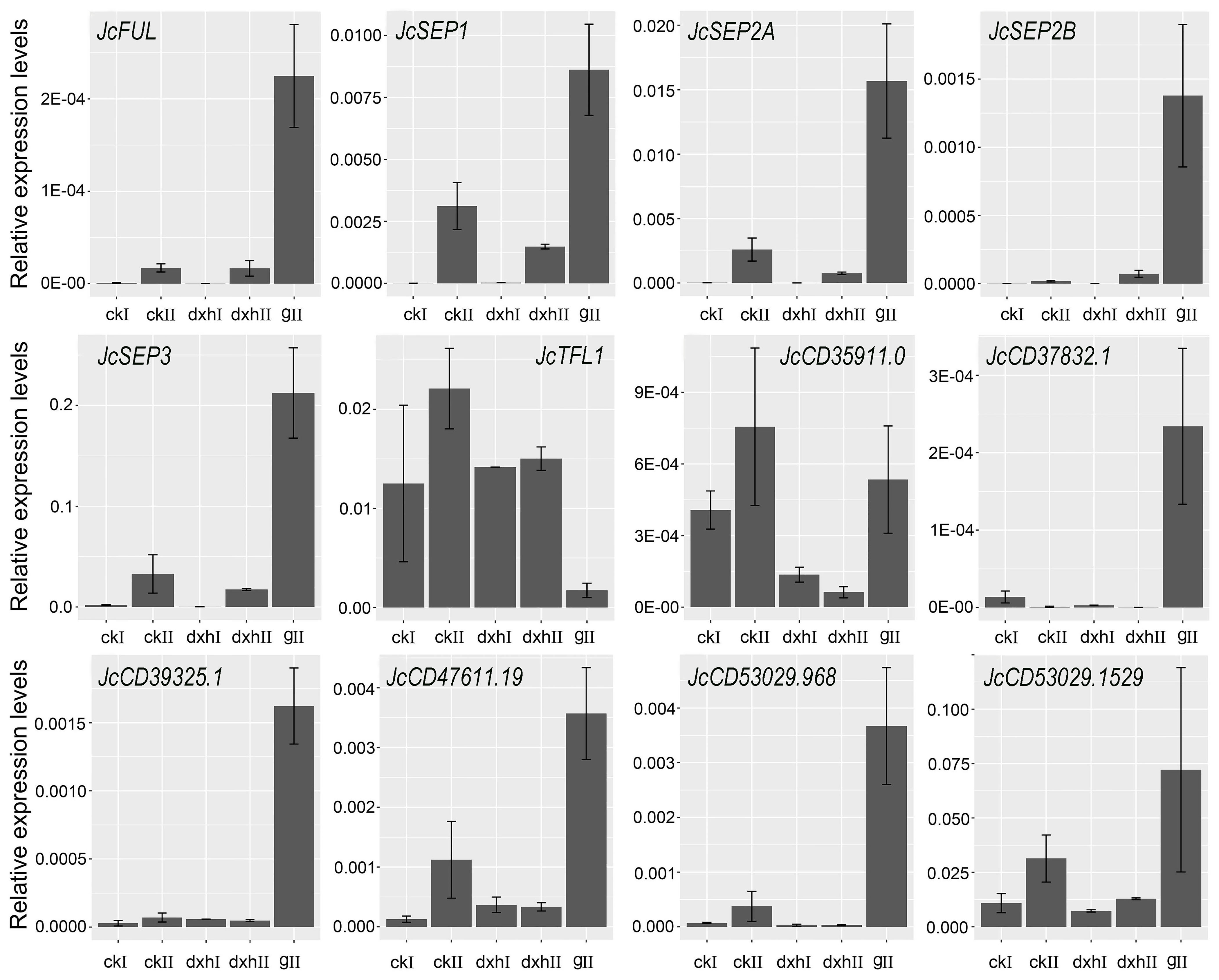

Supplement: Supplementary file 9 — Additional file 9. Validation of the expression profiles of 12 candidate genes by real-time qPCR. ckI and dxhI indicate the shoot tips of WT plants and dxh mutants; ckII, dxhII and gII indicate inflorescence buds of WT, dxh and g mutants, respectively. JcGAPDH was as the internal reference. The error bars represent SD (n = 3). [file 12870_2019_2069_MOESM9_ESM.jpg]

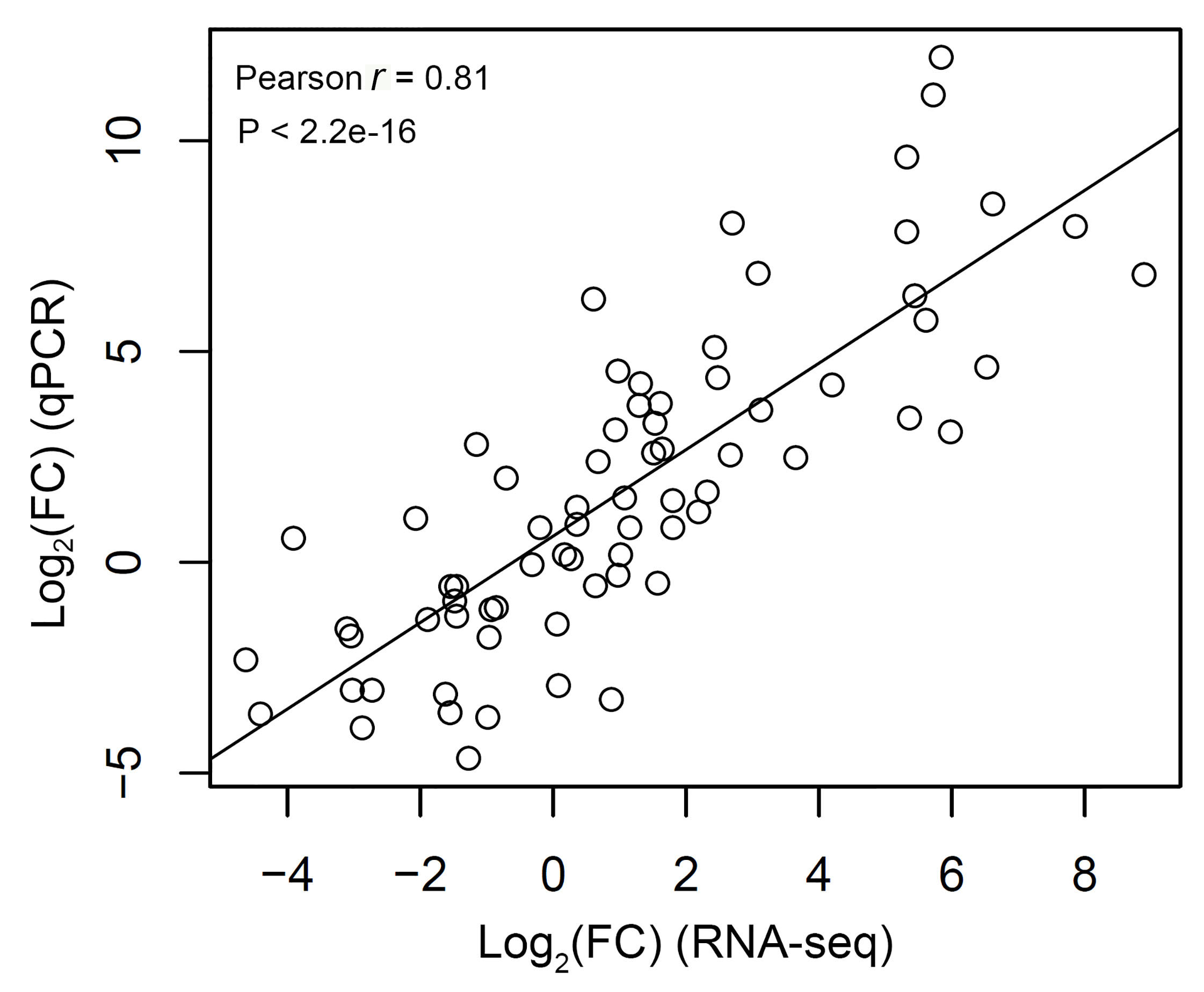

Supplement: Supplementary file 11 — Additional file 11. Correlation analysis between RNA-Seq and qPCR expression data of the genes shown in Additional file 9. The correlation analysis is performed with cor.test in R software. FC, fold change. [file 12870_2019_2069_MOESM11_ESM.jpg]

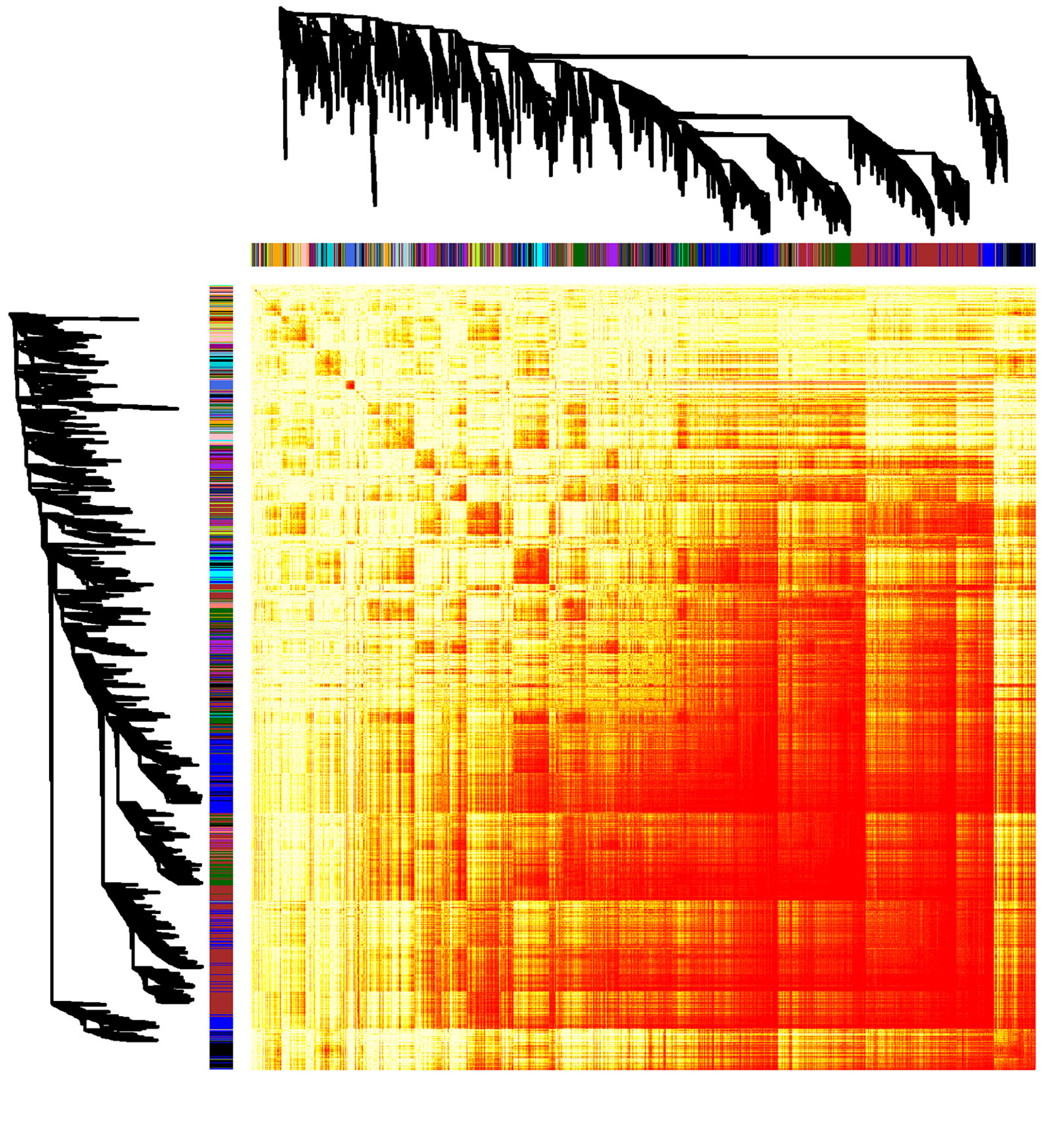

Supplement: Supplementary file 12 — Additional file 12. Weighted co-expression network and modules identified by WGCNA analysis. Each leaf in the hierarchical cluster tree represents one gene; each row and column of the heat map plot corresponds to one gene; in the heat map, light color indicate weak co-expression, and dark color indicates strong co-expression; twenty-two modules were labeled by different colors. [file 12870_2019_2069_MOESM12_ESM.jpg]

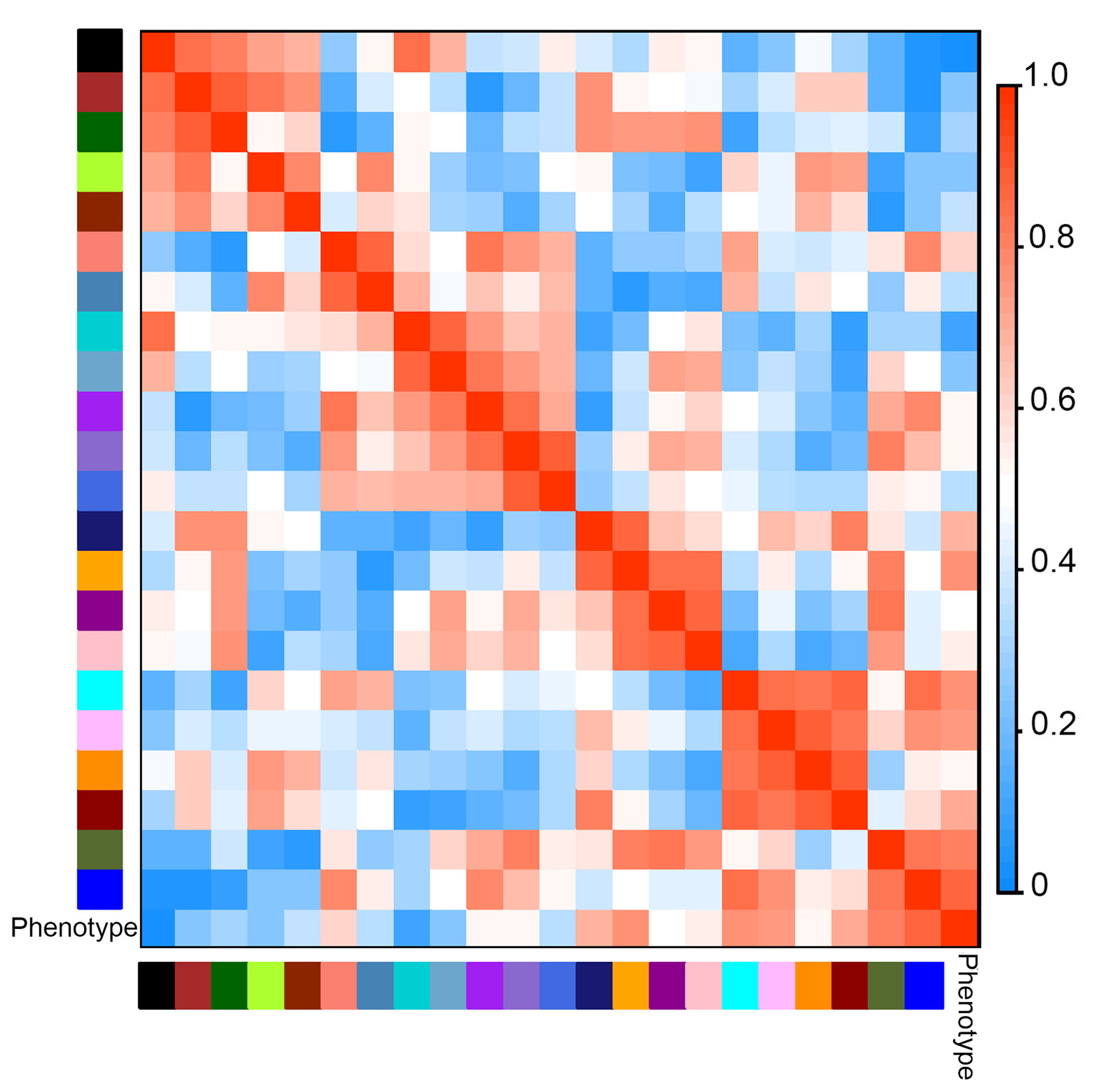

Supplement: Supplementary file 13 — Additional file 13. Correlation analysis of modules and phenotype traits. Each row and column represent one module; the red color represents high adjacency (positive correlation), and blue color represents low adjacency (negative correlation); red squares along the diagonal indicate the modules with similar expression patterns. [file 12870_2019_2069_MOESM13_ESM.jpg]

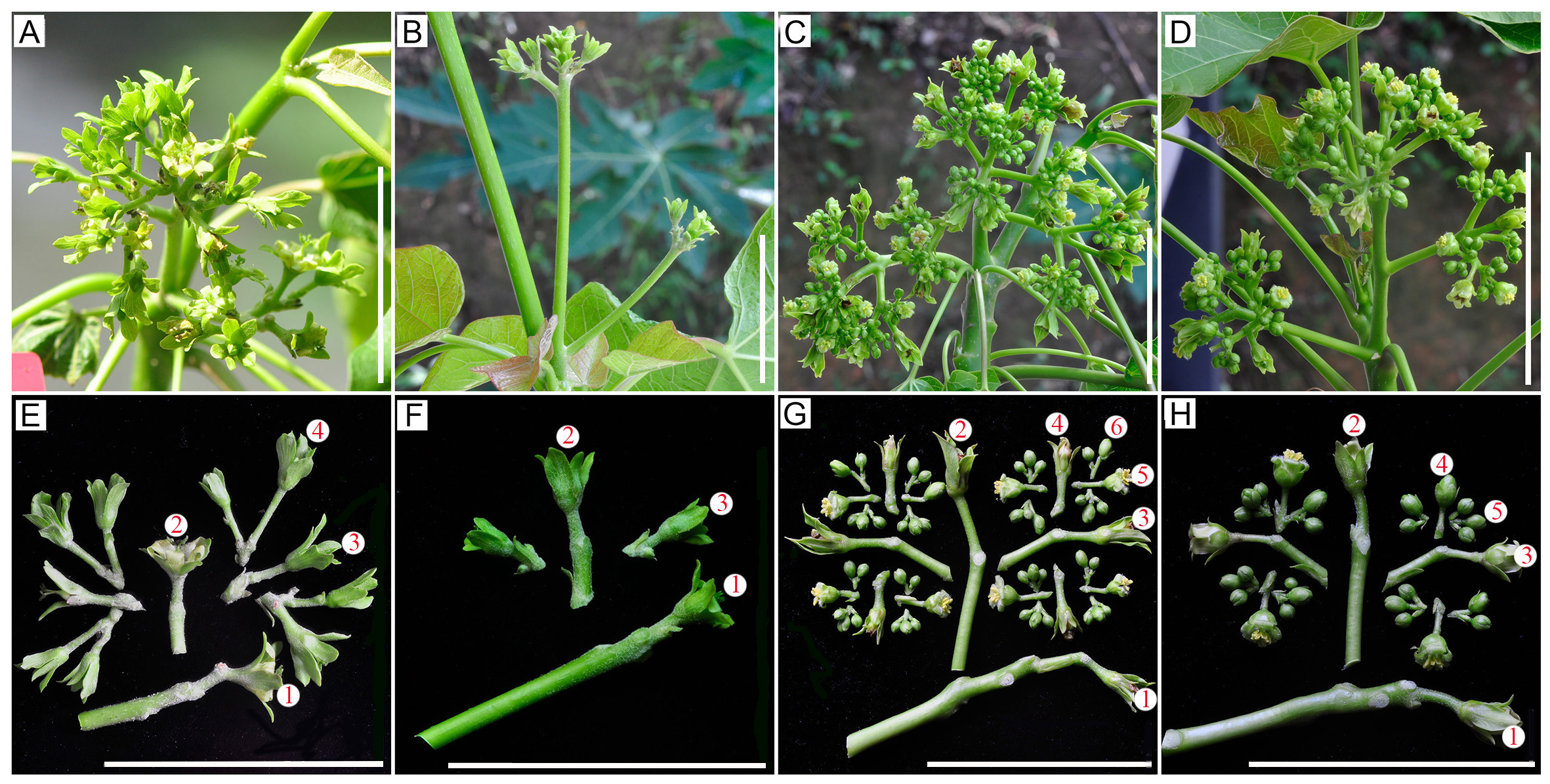

Supplement: Supplementary file 15 — Additional file 15. Application of 6-benzylaminopurine (6-BA) promotes inflorescence branching of g mutants and WT plants. (A)-(D) show the complete inflorescence, and (E)-(H) show the dissected inflorescence, respectively. (A) and (E) indicate an increased inflorescence branching treated with 6-BA and (B) and (F) display a normal inflorescence branching of g mutants with mock. (C) and (G) indicate an increase inflorescence branching treated with 6-BA and (D) and (H) display a normal inflorescence branching of WT plants with mock. The numbers in (E)-(H) represent different orders of inflorescence branching. Bar = 5.0 cm. [file 12870_2019_2069_MOESM15_ESM.jpg]
